# Catalytic acidic deep eutectic mixture for efficient and promising synthesis of quinazolinone and quinoxaline derivatives†

**DOI:** 10.1039/d5ra03346b

**Published:** 2025-07-21

**Authors:** Fatemeh Mohammad, Najmedin Azizi, Zohreh Mirjafari, Javad Mokhtari

**Affiliations:** a Department of Chemistry, Science and Research Branch, Islamic Azad University Tehran Iran; b Chemistry and Chemical Engineering Research Center of Iran P.O. Box 14335-186 Tehran Iran azizi@ccerci.ac.ir

## Abstract

A facilitated and economical protocol was designed to synthesize novel acidic deep eutectic mixture (ADEM) from urea/SbCl_3_/HCl as commercially available substrates for the first time. Urea acts as a hydrogen bond donor, SbCl_3_ serves as a Lewis acid catalyst, and HCl provides ionic conductivity and an acidic environment. The architectural chemical composition of acidic deep eutectic mixture was confirmed using EDX and FT-IR spectroscopy. This approach led to performing the quinazolinone synthesis with remarkable yield under moderate conditions in a short time and excellent functional group tolerance. Furthermore, in another reaction *via* a one-pot strategy, π-conjugated polycyclic quinoxaline frameworks were successfully synthesized. Moreover, ADEM demonstrated the capability of being reused and recycled for up to five terms while not decreasing the efficiency or impacting chemical performance regarding the reaction. Besides, the exploitation of inexpensive materials, time-saving reactions, and broad substrate range are prominent attributes of the designed procedure.

## Abstract 2

A novel acidic deep eutectic mixture (ADEM) from urea, SbCl_3_, and HCl was synthesized, enabling efficient, reusable catalysis for quinazolinone and quinoxaline derivatives under mild, economical conditions.

## Graphical abstract

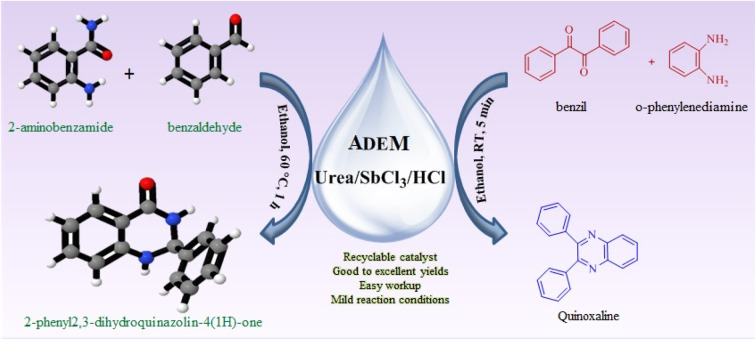

## 1. Introduction

The beating heart of human society development has been eco-friendliness from the beginning of the 21st century.^1^ By quickly boosting the global economy and the world population growth, modern chemistry can be one of the key players, tremendously relying on organic solvents.^2^ It should be noted that such solvents are mostly hazardous, toxic, and costly, producing waste by-products that harm health and safety and causing atmosphere pollution.^3,4^ The necessity of employing green chemistry and engineering concepts should not be overlooked to execute more sustainable and benign research.^5^ In this framework, the green advancement of the solvent is of fair importance, where sample pretreatment operations are mainly more benign and productive in organic chemistry.^6^ Accordingly, such solvents must satisfy particular prerequisites for being qualified as eco-friendly media, including traits such as reusability, biodegradability, cut-price, availability, and nontoxicity.^7–9^

According to the expressed reasons, ionic liquids (ILs) are a green classification of solvents based on their exceptional physicochemical features, which has grabbed increasing attraction in research studies.^10^ The salts in the liquid state are called ILs, which primarily contain organic cations with organic/inorganic anions at a low melting point of less than 373 K.^11^ Furthermore, they are considered eco-friendly solvents since their low vapor pressure makes them recyclable.^12^ On the negative side, the negligible biodegradability and the toxicity of some groups of ionic liquids give rise to challenges so that their industrial use would be restricted.^13^ Additionally, the synthesis processes of some ionic liquids are complicated and pricey, regarded as another issue. To meet the downside of ILs, emerging deep eutectic solvents (DESs) would be the most promising alternative approach in line with green chemistry purposes to face environmental as well as energy concerns.^14^

The first introduction of DES was reported by Abbott *et al.* in 2003.^15^ Generally, to produce DESs, two components with a proper molar ratio should be heated and stirred until the complete formation of a liquid mixture.^16^ DESs have been classified into five main groups according to their existing complexing agents, where the research top trends focus on the group Type III, when a hydrogen bond acceptor (HBA) interacts with a hydrogen bond donor (HBD), the resultant liquid mixtures of such a hydrogen bond interaction, which consists of two or more chemical compounds, are described as deep eutectic solvents (DESs).^17–19^ The hydrogen bonds creation as the output of such an interaction would lead to the charge relocation.^20,21^ In such a case, a eutectic mixture can be constructed where the temperature of the eutectic point illustrates a deep decline relative to that of a model liquid mixture.^22^ Additionally, such a eutectic mixture displays a remarkably lower melting point rather than its pure components.^23–25^ DESs are fascinating solvents having multiple notable attributes, such as high thermal and chemical stability, poor vapor pressure, remarkable conductivity, wide liquid range, high purity, intrinsic biodegradability, non-flammability, non-hazardous, and a wide chemical window without producing waste material. Moreover, the capacity of DESs to be fabricated on large and industrial scales at a rather low expense is rooted in their facile and cut-price synthesis procedure.^26–28^ Besides, DES systems are productively suitable for the mass fabrication of state-of-the-art functional materials, which brings about the extensive exploitation of deep eutectic solvents ascribed to such outstanding features in vast schemes.^29–31^

The transition from ionic liquids (ILs) to DESs presents a familiar narrative where past mistakes can resurface if not heeded. To prevent history from repeating itself, it is crucial to address potential environmental impacts, toxicity concerns, sustainability issues, regulatory oversight, and public perception early on in the development of DESs.^32^ Learning from the pitfalls of ILs, stakeholders must prioritize sustainability, safety, and transparency to ensure a more balanced and responsible evolution of DESs as alternatives in the chemical industry. The greenness of DESs had been a subject of ongoing research and debate. Studies have highlighted both the potential environmental benefits and concerns associated with DESs, emphasizing factors such as their biodegradability, toxicity, and energy consumption during production.^33^

Ring-fused polycyclic configurations are tremendously present in diverse natural materials and pharmaceutical molecules. Subsequently, building such configurations from easily approachable substrates has gone mainstream in organic synthesis among research subjects.^34–36^ Therefore, it should be noted that the investigation of the N-containing heterocyclic compounds synthesizing has always been an appealing topic for synthetic chemists.^37^ Among them, two groups of heterocyclic compounds, quinazolinone and quinoxaline, have received more attention thanks to their extraordinary attributes.^38^ The quinazolinone derivatives have arisen as an intriguing nitrogen-containing scaffold in the scope of drug discovery, chemical exploration, and medication development. Compounds holding such a framework reveal a broad range of biological and pharmaceutical activities but are not restricted to antimicrobial, antimalarial, anti-inflammatory, antihypertensive, and anticancer properties.^39^ On the other hand, quinoxaline recognized as benzopyrazine, which is considered an exceptional multi-nitrogen heterocyclic compound.^40–42^ The current state of the art in the preparation of quinazolinone and quinoxaline derivatives involves a diverse range of synthetic methods aimed at enhancing efficiency, selectivity, and sustainability. Innovative strategies include the use of catalytic processes, green solvents, and novel reaction conditions to improve yields and reduce environmental impact in quinazolinone synthesis.^43–45^ Similarly, advancements in quinoxaline derivative synthesis focus on transition-metal catalysis, photochemical reactions, and cascade reactions to access diverse structural motifs with enhanced efficiency and selectivity. These developments underscore a shift towards sustainable and efficient methodologies in organic synthesis, reflecting the ongoing efforts to optimize the preparation of these important heterocyclic compounds in modern organic chemistry research.^46,47^

Based on the above assumptions, we are motivated to develop urea/SbCl_3_/HCl as a new and simple ADEM as catalyst and solvent for the synthesis of N-containing heterocyclic compounds. The true worth of organic transformation practices becomes brighter when the entire set of green chemistry principles is integrated into a single pathway to achieving extraordinary yields with minimal harmful repercussions for the environment. Synthesis of quinazolinone and quinoxaline derivatives was taken into account as one of the main objectives of this study owing to their great value in the pharmaceutical industry. The novel ADEM as a catalyst assisted in conducting such reactions with high yields at short times and mild reaction conditions.

## 2. Experimental section

### 2.1. Materials and equipment

Starting materials and reagents, including aldehyde derivatives (≥98%), 2-aminobenzamide (≥98%), *o*-phenylenediamine (≥98%), and benzil (≥98%), were sourced from Merck. Analytical grade SbCl_3_ (98%) was obtained from Sigma-Aldrich, while urea (98%), HCl (37%), and organic solvents like ethanol (96%), methanol (99%), toluene (99%), and ethyl acetate (98%) were procured from a local company in Iran. The reactions were carried out in test tube vials with a septum, and product characterization was conducted by comparing melting points with authentic samples using a Buchi 535 melting point apparatus. Fourier transform infrared (FT-IR) spectra were recorded on a Bruker Vector-22 FT-IR spectrometer with KBr disks. Energy-dispersive X-ray spectroscopy (EDS) was performed using a scanning electron microscope (VEGA3 TESCAN) at an operating voltage of 20 kV, Zeiss-Sigma VP model. ^1^H-NMR and ^13^C NMR spectra were obtained on 500 MHz and 125 MHz spectrometers using CDCl_3_ and DMSO-d_6_ as the solvent, respectively, with chemical shifts reported in parts per million (ppm) relative to TMS. Mettler Toledo DSC instruments were employed for DSC analysis, and the rate of temperature change was 5 °C min^−1^ in the temperature region 0 °C to 300 °C. Water Content was Determined by Karl Fischer Analysis using 684KF Coulometer Metrohm.

### 2.2. Preparation of acidic deep eutectic mixture

**Fig. 1:**
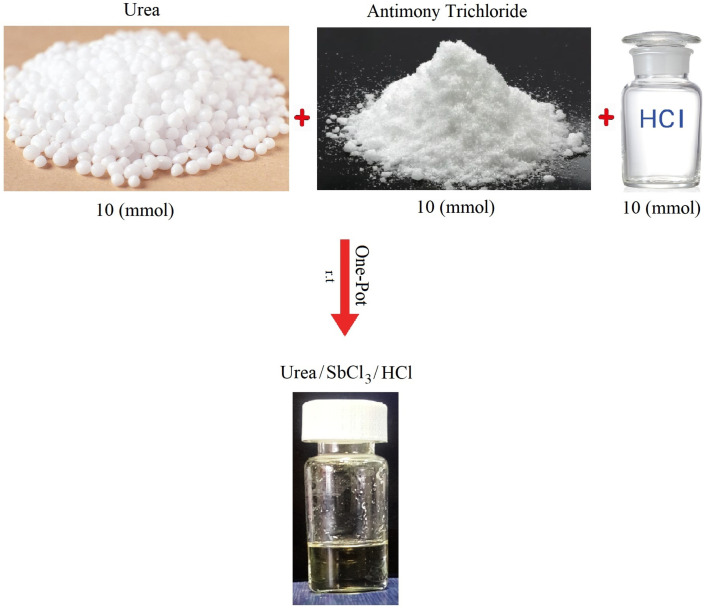
Preparation of ADEM.

The ADEM was synthesized by a direct and facile procedure. In a reaction container, urea (10 mmol), SbCl_3_ (10 mmol), and HCl 37% (10 mmol) were added respectively, and then the mixture was stirred at room temperature until reaching a colorless liquid, which showed that ADEM was formed (Fig. 1).

### 2.3. General protocol for the synthesis of 2,3-dihydroquinazolin-4(1*H*)-one *via* acidic deep eutectic mixture

In a test tube with a magnetic stirring bar, 2-aminobenzamide (0.5 mmol), benzaldehyde (0.5 mmol) and ADEM (50 mg) and ethanol (1 mL) were added. Subsequently, the reaction mixture was heated at 60 °C for 60 min, where the reaction advancement was scanned using TLC. After completion of the reaction, ethanol was removed and ethyl acetate (10 mL) and water (10 mL) were added. Then, two phases were separated and ethyl acetate was removed by Rotary evaporation. The crude products were recrystallized *via* ethanol and diethyl ether performed to isolate analytical pure 2,3-dihydroquinazolin-4(1*H*)-one derivatives.

### 2.4. General protocol for the synthesis of quinoxaline *via* acidic deep eutectic mixture

A mixture of *o*-phenylenediamine (0.5 mmol), benzil (0.5 mmol), ADEM (50 mg), and ethanol (1 mL) was wadded in a test tube. Then, this reaction mixture was stirred at room temperature for 5 min, where the reaction advancement was scanned through TLC. After completion of the reaction, ethanol was removed and ethyl acetate (10 mL) and water (10 mL) were added to the reaction mixture. Then, two phases were separated and ethyl acetate was removed by Rotary evaporation. The crude products were recrystallized *via* ethanol and diethyl ether performed to isolate analytical pure quinoxaline derivatives.

## 3. Result and discussion

### 3.1. Characterization of synthesized acidic deep eutectic mixture

We have designed, prepared, and identified ADEM as a unique class of acidic deep eutectic mixture. This novel system was characterized by Fourier transform infrared (FT-IR), energy-dispersive X-ray (EDX) DSC, and NMR spectroscopy.

#### 3.1.1. Fourier transform infrared spectroscopy (FT-IR)

**Fig. 2:**
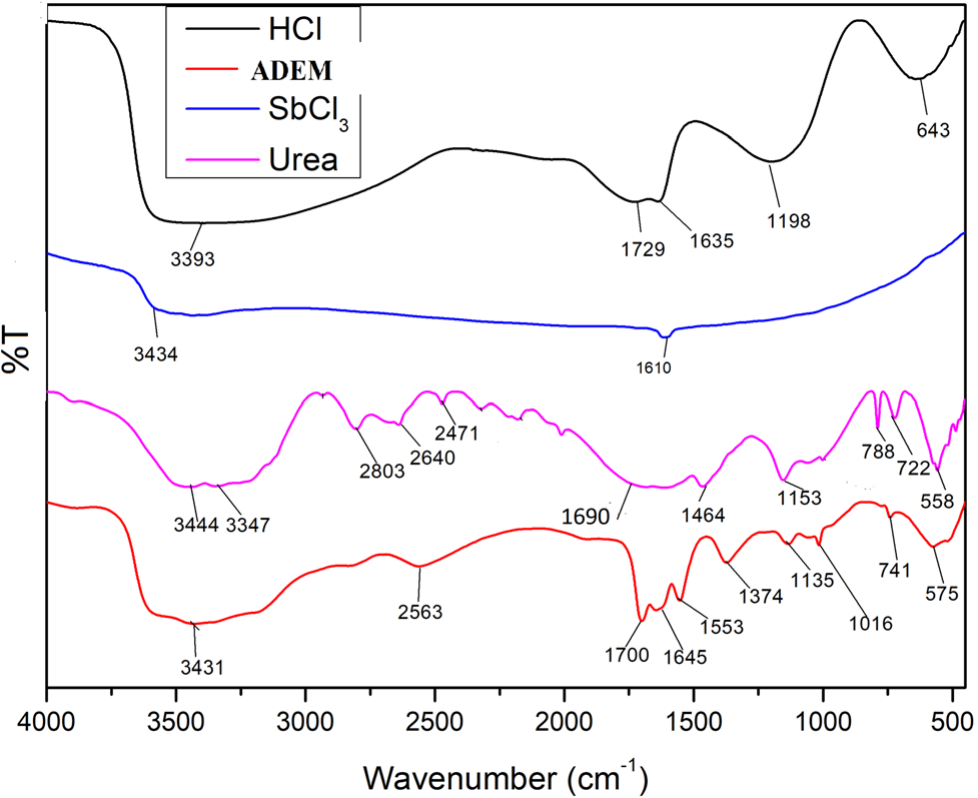
The FT-IR spectrum of ADEM.

A new acidic deep eutectic mixture were successfully synthesized and identified *via* FT-IR spectroscopy and results were shown in Fig. 2 which depicts evident absorption bands. The broad bands for O–H and N–H stretching vibrations were detected at 3431 and 3580 cm^−1^, respectively. Characteristic bands stemmed from the C–N bending and stretching vibrations were observed at 1553 and 1135 cm^−1^, respectively. The stretching vibration of C=O was spotted by a characteristic band at 1645 cm^−1^, with a scant shift. Such a change distinguishes the construction of coordination bonds between oxygen atoms in the C=O group and the existing Sb^3+^ ions in the acidic deep eutectic mixture. The Characteristic peaks marked in the IR area below 800 cm^−1^ can be representative of the stretching state of H–Cl and urea. Furthermore, FTIR studies of ADEM did not show strong peaks around 830 cm^−1^, which are typically associated with Sb–O–Sb bonds. Moreover, the signals in the 550–650 cm^−1^ region, corresponding to Sb–O vibrations, were also weak or absent. These results suggest the lack of significant Sb–O bonding in the ADEM structure.

#### 3.1.2. Energy dispersive X-ray (EDX)

**Fig. 3:**
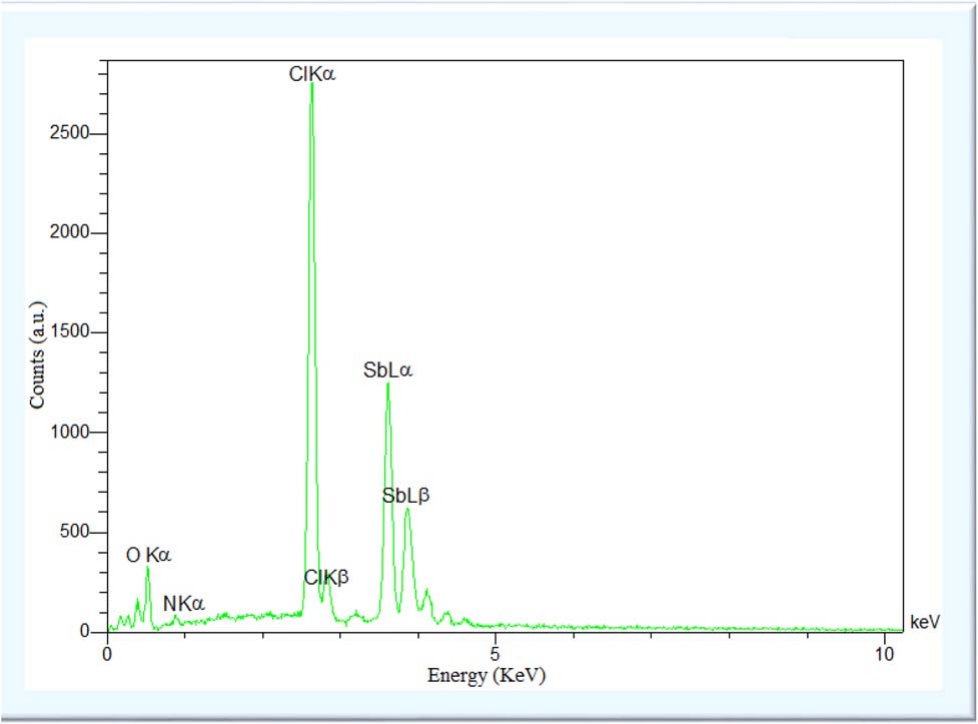
EDX analysis of ADEM.

Energy-dispersive X-ray spectroscopy (EDX) was deployed to analyze the elemental composition corresponding to ADEM. Fig. 3 presents the EDS pattern, affirming the attendance of O, Sb, and Cl. The existence of such elements in the EDS pattern gives convincing proof of the effective embedding of Sb in the as-prepared system. The presence of Sb in the spectrum is due to the efficient incorporation of SbCl_3_.

**Fig. 4:**
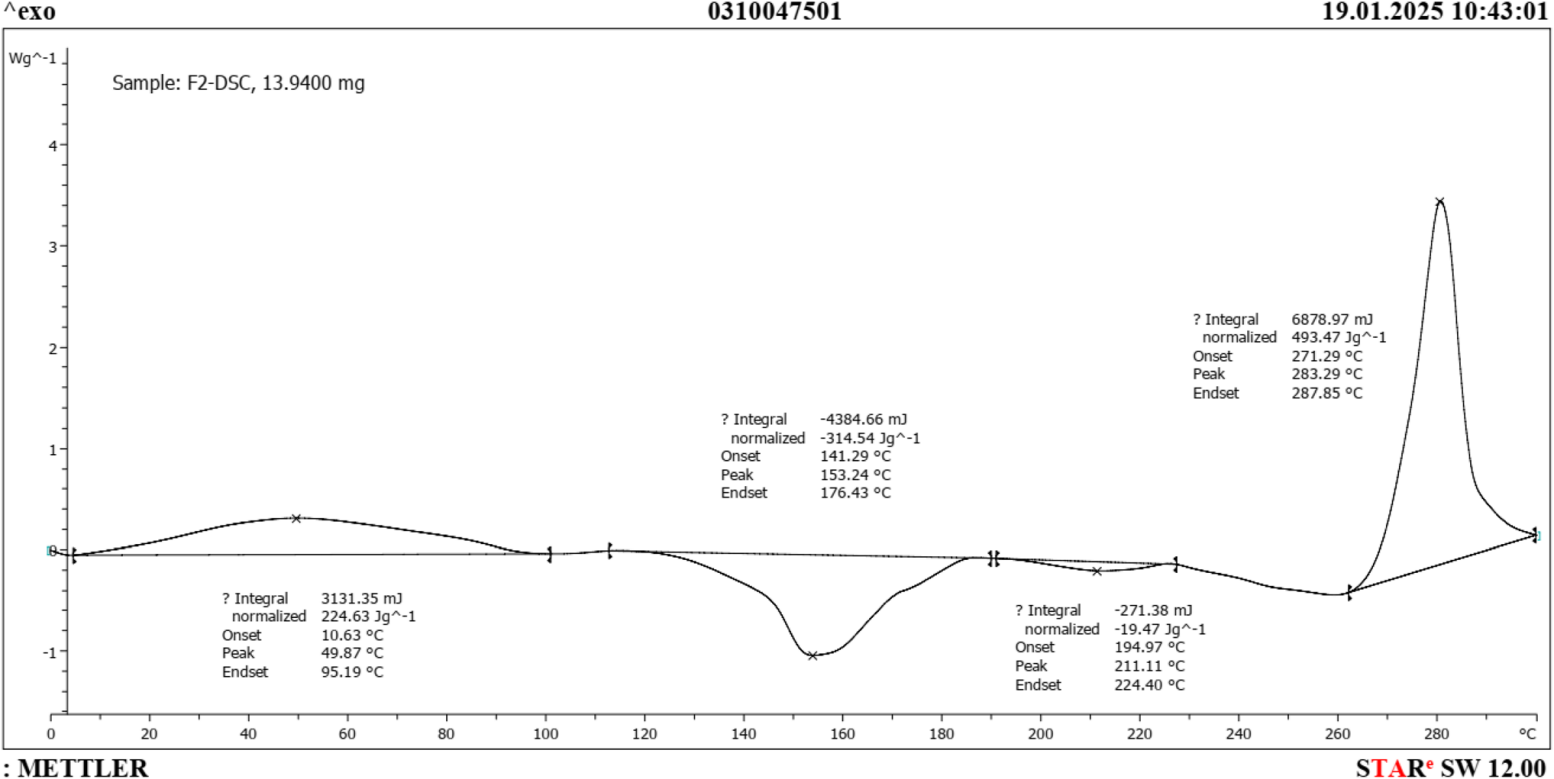
DSC analysis of ADEM.

The DSC analysis provided information about the thermal behavior of the synthesized compounds, including details such as melting points, phase transitions, and heat capacities. The DSC analysis was conducted with a temperature change rate of 5 °C min^−1^ within the temperature range of 0 °C to 300 °C (Fig. 4). The DSC diagram revealed three distinct phase changes, specifically involving melting, crystallization, and boiling of the ADEM.

**Fig. 5:**
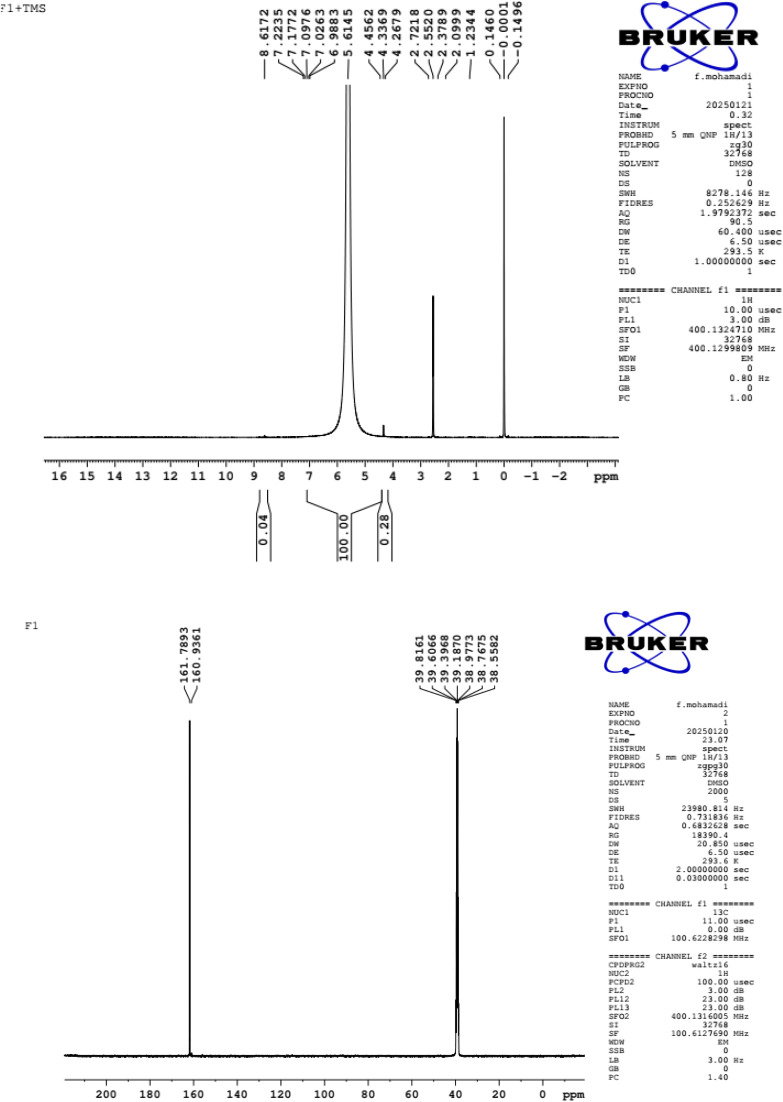
^1^HNMR and ^13^C NMR spectrum of ADEM.

NMR spectroscopic techniques have been utilized to evaluate the molecular structure of acidic deep eutectic mixture composed of SbCl_3_, urea, and aqueous HCl, with the results shown in Fig. 5. All signals in the ^1^H and ^13^C NMR spectra (Fig. 5) have been assigned. In the ^1^H NMR spectrum recorded at 500 MHz and 298 K, the hydrogen signals from water, urea, and HCl were observed as a broad peak in the chemical shift range of *δ* 5.00–6.00 ppm. This peak was slightly shifted compared to the normal urea signal at 6.12 ppm. Furthermore, the ^13^C NMR spectrum obtained at 125 MHz exhibited only two peaks corresponding to the carbonyl carbons of urea. These peaks appeared at 160 and 161 ppm, showing a shift slightly relative to the urea-based DES signal at 161.9 ppm.^48^

Water's impact on the physicochemical properties of ADEM is a current research focus. Through Carl Fisher analysis and DSC spectroscopy, the intermolecular interactions in water-ADEM were studied. The Carl-Fisher analysis revealed that the water content in the acidic system was 16%. Furthermore, various concentrations of HCl (ranging from 15% to 37%) as water sources were used to prepare ADEM, to investigate the impact of water content. It was found that within the range of 5–23% (w/w) water, the eutectic characteristics were maintained, resulting in the formation of homogeneous liquids with varying viscosities. However, with an increase to around 25% (w/w) water, the strong interactions among the three ADEM components weakened gradually, causing the eutectic mixture to decompose into two phases. Moreover, reducing the water content to 5% under vacuum conditions led to the transformation of ADEM into a semisolid state. In the ADEM system, water molecules played a crucial role in enhancing hydrogen bonding with NH and OH donors, as well as solvated ADEM (Fig. S16 in ESI†).^49^

### 3.2. Evaluation of synthesized acidic deep eutectic mixture activity for the synthesis of quinazolinone and quinoxaline derivatives

Following the successful synthesis and identification of ADEM, its catalytic activity was investigated in the synthesis of substituted quinazolinone and quinoxaline. This study aimed to design a benign and recyclable method for preparing quinazolinone and quinoxaline derivatives utilizing easily accessible substrates. The catalytic system of this work proposed a green and selective technique, using low-cost and non-hazardous starting materials and reactants.

First, the synthesis of quinazolinone was investigated; accordingly, to achieve this goal, a comprehensive evaluation of reaction conditions in the model reaction was optimized.

2-Aminobenzamide and benzaldehyde were employed in the model reaction to optimize the condition of quinazolinone synthesis, and the obtained results are listed in Table 1. First, the amount of catalyst was optimized, and the results showed that 50 mg of ADEM gives excellent yields for the quinazolinone synthesis. Additionally, to evaluate the feasibility of the reaction without acidic deep eutectic mixture, the model reaction was carried out without exploiting ADEM as a catalyst, leading to low efficiency (Table 1, entries 1–6). It is apparent from Table 1 that the selection of solvent and temperature are two deciding factors for the evaluation of the reaction yield in the quinazolinone synthesis. In the next step, optimizing the used solvent was taken into account. For the synthesis of quinazolinone, four solvents including ethanol, methanol, chloroform, and toluene were used in the same conditions, and the results illustrated that using 1 mL of ethanol as a solvent would bring about the best efficiency (Table 1, entries 1). It should be noted that if pure urea, HCl, and urea/HCl are individually employed as the catalysts, it would give lower yields of the expected product (Table 1, entries 10–12). More precisely, the application of ADEM with a ratio of 1 : 1 : 1 would lead to the best output, implying that the compelling reason behind obtaining higher yields is the collaborative mixture of the components in the ADEM. The optimal conditions in all cases for the synthesis of quinazolinone included the use of 0.5 mmol of both 2-aminobenzamide and benzaldehyde as reactants. Accordingly, entry 1 indicates the optimal reaction in which employing 50 mg ADEM as the catalyst at the reaction temperature of 60 °C gives the product to a yield of 97% within one hour using 1 mL of ethanol as the solvent.

**Table 1:** Optimization of the reaction conditions in the straightforward synthesis of 2,3-dihydroquinazolin-4(1*H*)-one *via* ADEM

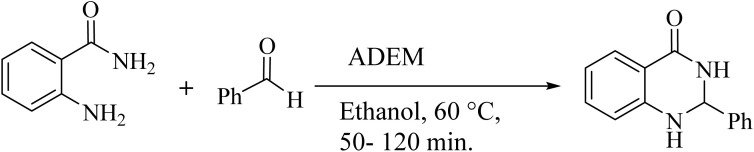				
Entry	DES (catalyst)	Catalyst (mg)	Solvent (1 mL)	Yield^a^ (%)
1	ADEM	**50**	**Ethanol**	**97**
2	ADEM	40	Ethanol	83
3	ADEM	30	Ethanol	75
4	ADEM	20	Ethanol	60
5	ADEM	10	Ethanol	54
6	ADEM	0	Ethanol	40
7	ADEM	50	MeOH	84
8	ADEM	50	CHCl_3_	30
9	ADEM	50	PhCH_3_	35
10	Urea	50	Ethanol	45
11	HCl	50	Ethanol	50
12	Urea:HCl	50	Ethanol	60
13	SbCl_3_	50	Ethanol	68
14	Urea:SbCl_3_	50	Ethanol	74
15	SbCl_3_:HCl	50	Ethanol	81
16	ADEM	100	—	74
17	ADEM	200	—	86
18	ADEM	400	—	90
19	ADEM	600	—	97
20	ADEM	50	Water	79
21	ADEM	50	Water/ethanol (1 : 1)	91

a Isolated yields.

To investigate the generalization of such a model reaction, several 4(3*H*)-quinazolinones were fabricated, applying various aromatic aldehydes subjected to optimal reaction conditions. Additionally, the reactions between aromatic aldehydes with components bearing electron donating/withdrawing groups were productively conducted for each state, delivering the desired products with outstanding yields in time-saving reactions in which the aldehyde type had no tangible impact on the reaction (Table 2).

**Table 2:** One-pot synthesis of various substituted 2,3-dihydroquinazolin-4(1*H*)-one from different benzaldehydes *via* ADEM under optimum conditions

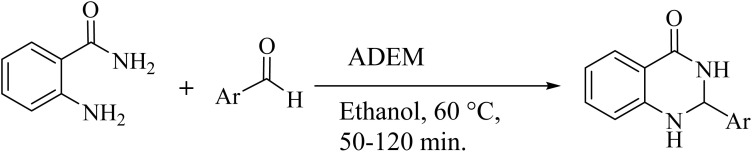						
Entry	R	Product	Time (min)	Yield (%)	M.p (°C)	M.p (°C)
					Found	Reported
1	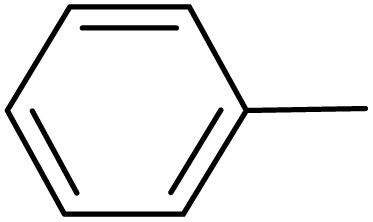	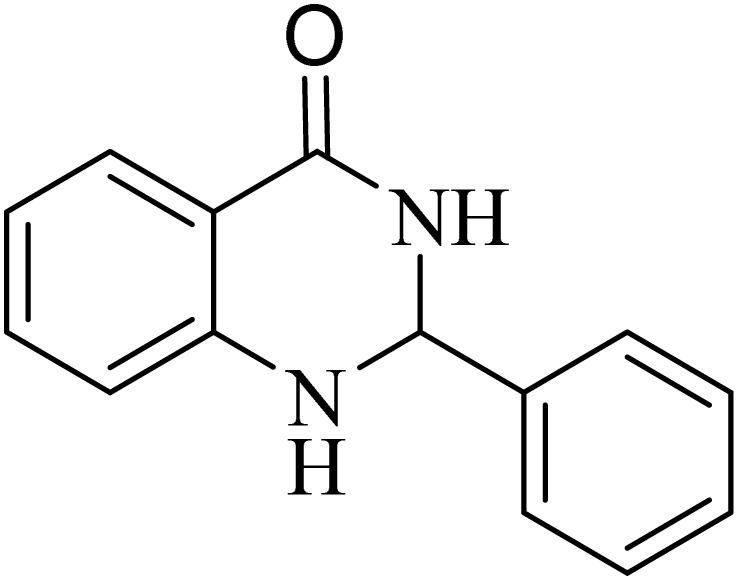	60	97	218–219	220–222 (ref. 51)
2	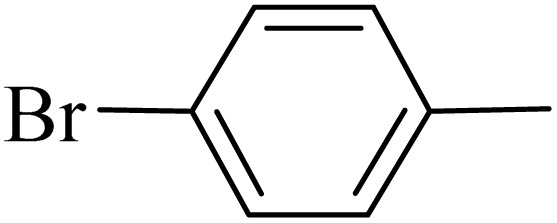	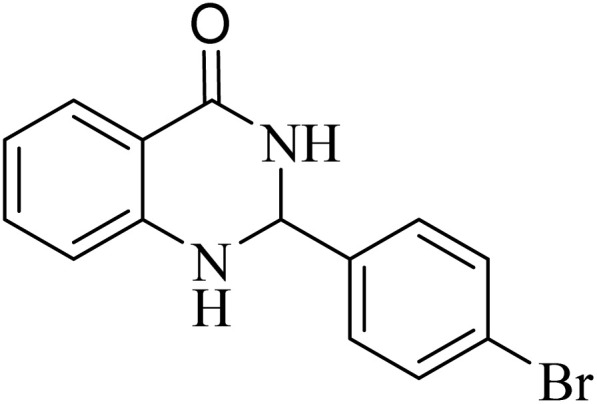	60	86	197–199	195–197 (ref. 51)
3	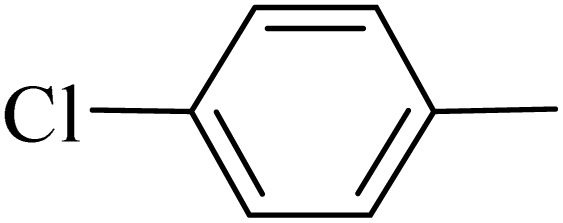	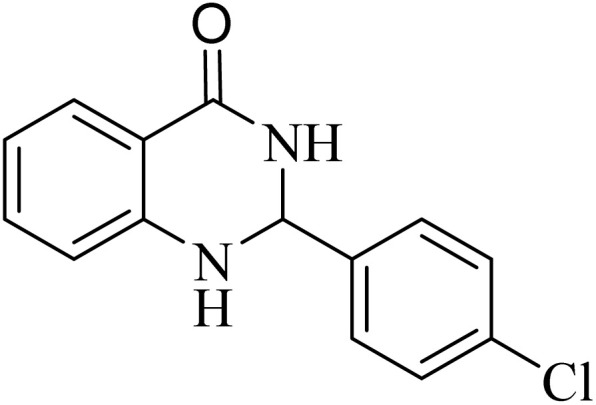	60	96	207–209	205–206 (ref. 51)
4	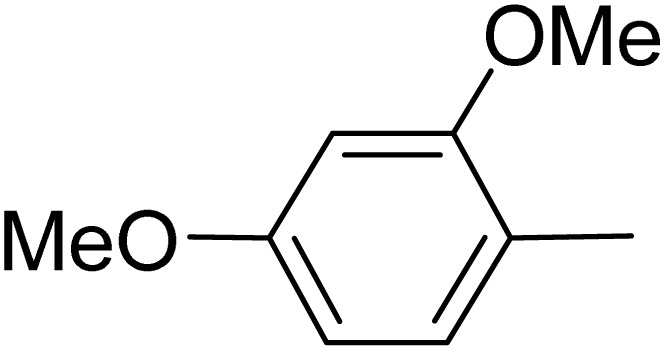	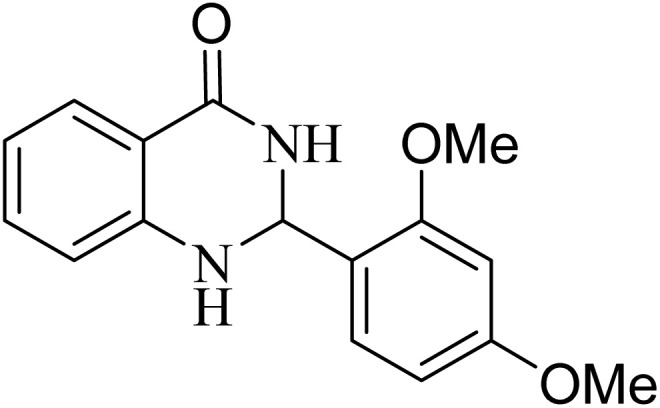	110	81	189–190	186–188 (ref. 51)
5	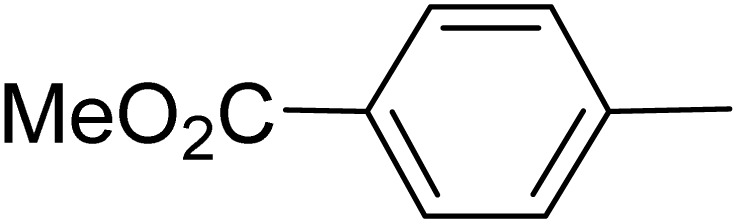	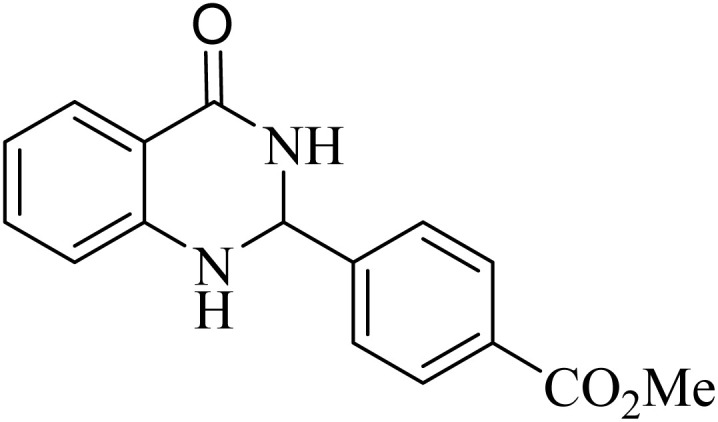	60	94	217–219	218–220 (ref. 51)
6	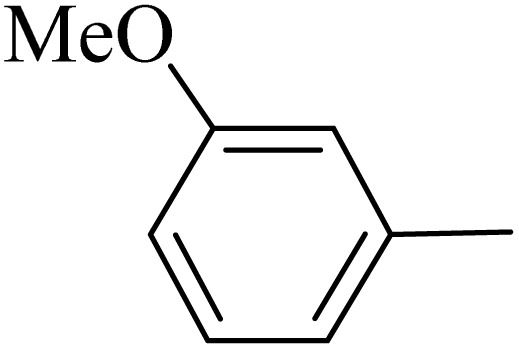	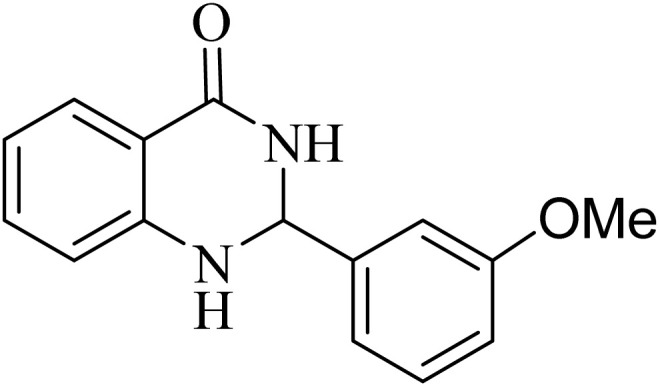	60	86	224–226	226–227 (ref. 51)
7	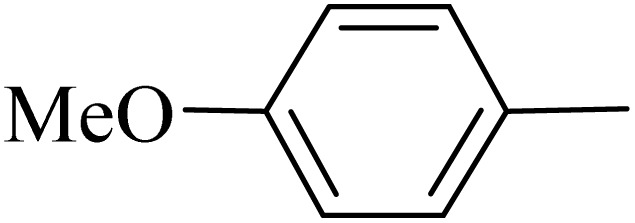	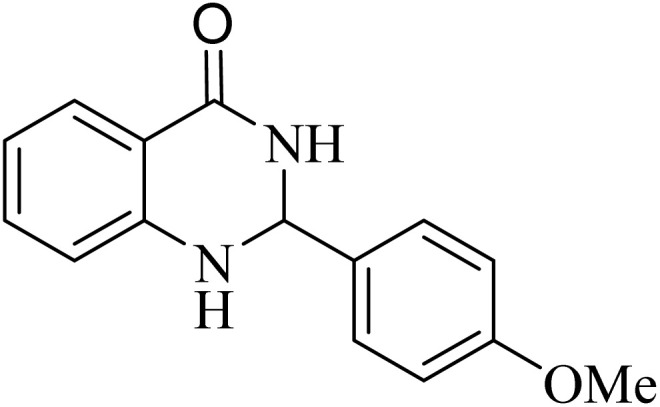	60	87	183–185	185–186 (ref. 43)
8	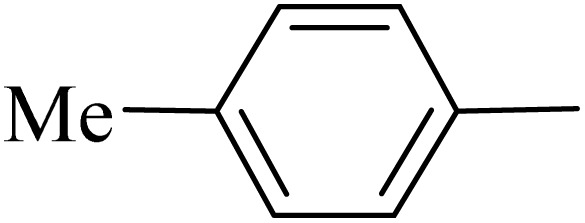	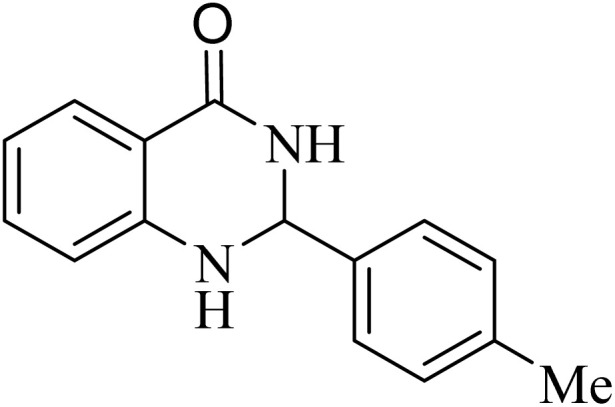	60	94	223–224	220–221 (ref. 43)
9	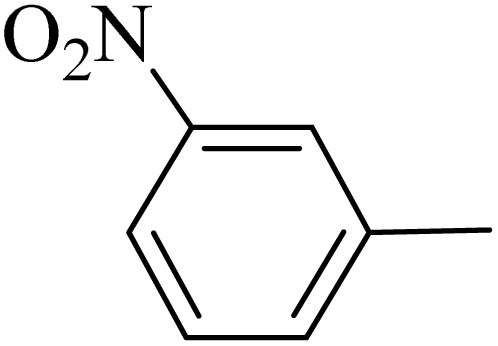	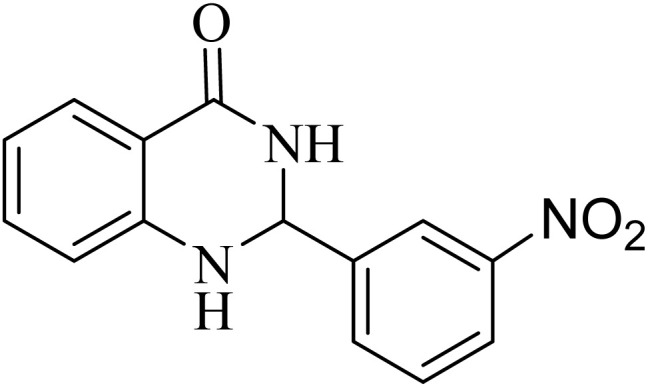	50	92	211–213	216–217 (ref. 60)
10	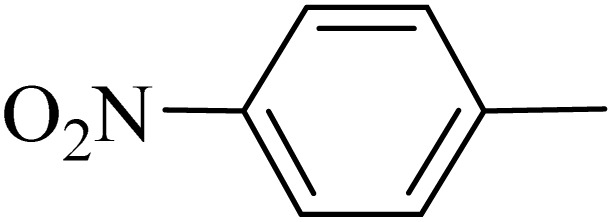	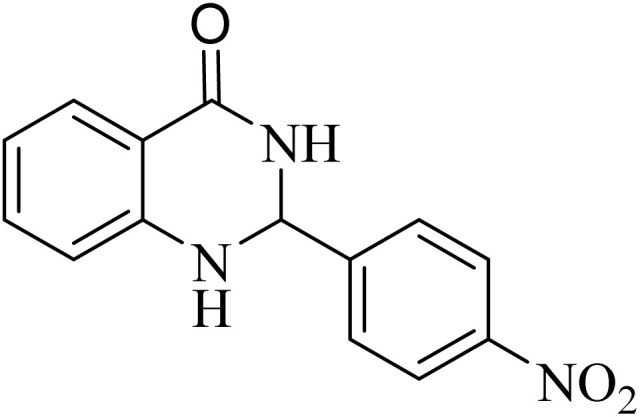	90	88	198–199	198–200 (ref. 43)

The use of ADEM in the corresponding reaction was compared to the other catalysts described in previous publications in order for supplementary exploration of its catalytic activity. The results are outlined in Table 3, proving the privilege of this procedure rather than the expressed techniques due to its excellent efficiency, short reaction time, gentle and environmentally friendly conditions, great yield, and catalyst reusability.^50–59^

**Table 3:** Comparative catalytic activity of ADEM with other reported catalysts in synthesis of quinazolinone

Entry	Catalyst	Conditions	Time	Yield (%)	Ref.
1	[bbim]Br as IL	120 °C	3.5 h	92	53
2	ChSO_3_HCl	H_2_O (1 mL), rt	1 h	95	51
3	Cu(NO_3_)_2_·3H_2_O	CH_3_CN, 80 °C	9 h	93	55
4	TBHP	H_2_O, 110 °C	16 h	90	56
5	Tyrosinase	DMSO, air, 100 °C	20 h	73	57
6	Ru(bpy)_3_Cl_2_·6H_2_O/9-fluorenone	CH_3_OH, white light-emitting diode (LED) 18 W in air	2 h	86	58
7	—	C_anode_/Al_cathode_/AcOH/MeOH/rt	3–5 h	86	59
8	ADEM	EtOH, 60 °C	1 h	97	This work

In the next step, the synthesis of quinoxaline was evaluated to execute a comprehensive exploration. *o*-Phenylenediamine and benzil were utilized in the model reaction to optimize the condition of quinoxaline synthesis and the obtained results are list in Table 4. First, the amount of ADEM was optimized exhibiting that the best reaction yield is acquired when 50 mg of catalyst is involved in the quinoxaline synthesis. As illustrated in Table 4 (entries 1–5) by decreasing the amount of the catalyst from 50 mg to 10 mg, the obtained yields declined from 98% to 63% respectively. Furthermore, by eliminating the catalyst, the final yield decreased dramatically (50%, entry 6). In the next phase, optimizing the exploited solvent was taken into account. For the synthesis of quinoxaline, four solvents including ethanol, methanol, dimethyl sulfoxide, and acetonitrile, were operated in the same conditions, and the outcomes depicted that utilizing 1 mL of ethanol (entry 1) would lead to the highest yield when compared to entries 7–9. Undoubtedly, if urea, HCl, SbCl_3_ and urea/HCl are individually used as the catalysts, lower yields of the considered product were obtained (Table 4, entries 10–12). More specifically, the application of urea/SbCl_3_/HCl with a ratio of 1 : 1 : 1 would lead to the best result, signifying that attaining higher yields is rooted in the cooperative mixture of the components in the ADEM. The optimal conditions for the synthesis of quinoxaline included the use of 0.5 mmol for both *o*-phenylene diamine and benzil as reactants in addition to 1 mL of ethanol as a solvent while employing 50 mg ADEM as the catalyst at room temperature which leads to a yield of 98% within 5 minutes.

**Table 4:** Optimization of the reaction conditions in the straightforward synthesis of quinoxaline *via* ADEM

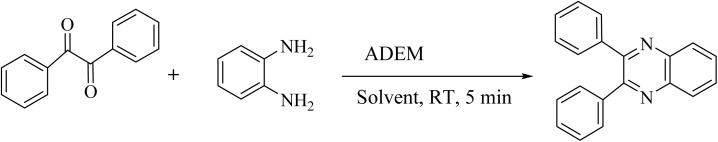				
Entry	DES (catalyst)	Catalyst (mg)	Solvent (1 mL)	Yield^a^ (%)
**1**	ADEM	**50**	**Ethanol**	**98**
2	ADEM	40	Ethanol	90
3	ADEM	30	Ethanol	82
4	ADEM	20	Ethanol	70
5	ADEM	10	Ethanol	63
6	ADEM	0	Ethanol	50
7	ADEM	50	MeOH	89
8	ADEM	50	DMSO	81
9	ADEM	50	CH_3_CN	92
10	Urea	50	Ethanol	55
11	HCl	50	Ethanol	60
12	Urea:HCl	50	Ethanol	70
13	ADEM	50	Water	67
14	ADEM	50	Water/ethanol (1 : 1)	83

a Isolated yields.

By utilizing diverse derivatives of *o*-phenylenediamines and benzil under optimal conditions, different quinoxaline substituents were successfully synthesized in the existing acidic deep eutectic mixture as a catalyst to appraise the extent and versatility of catalyst capability in quinoxaline synthesis. Overall, the results depicted that *o*-phenylenediamines, whether reacted with electron-donating or withdrawing groups, can provide favorable products. However, the reaction of *o*-phenylenediamines with withdrawing substitutions needed a more prolonged time for fulfillment with even lower yields for some circumstances (Table 5, entries 2, 5, and 7). On the other hand, the reaction of acenaphthylene-1,2-dione as a reactive α-diketone with *o*-phenylenediamines and ethylenediamines completed quickly acquiring the expected products in superior yields (Table 5, entries 10–13).^60–63^

**Table 5:** Quinoxaline derivatives from different 1,2-diamines and 1,2-diketones *via* ADEM under optimum conditions

Entry	Diamine	Benzil	Product	Time	Yield (%)	M.p (°C) [ref.]
1	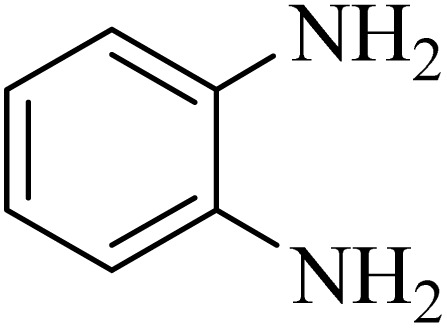	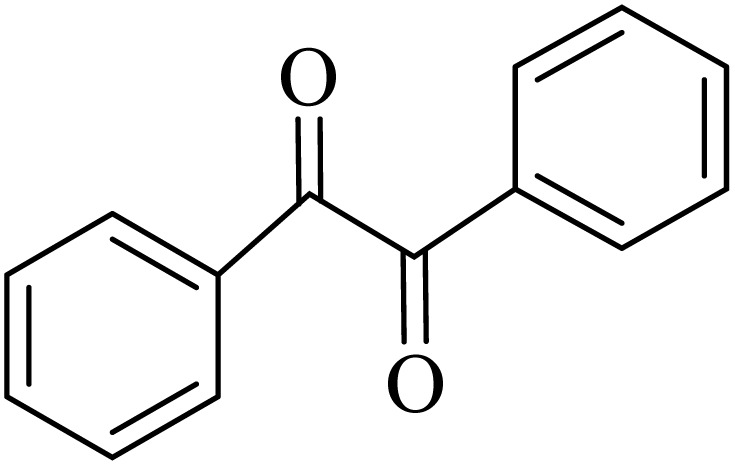	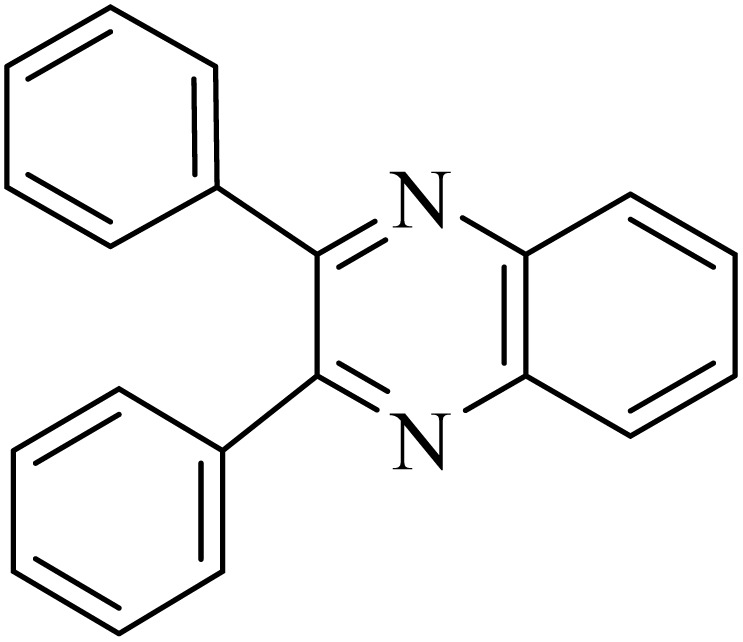	5	98	128–129 (ref. 60)
2	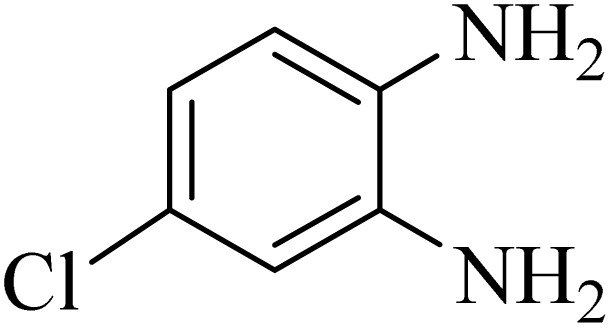	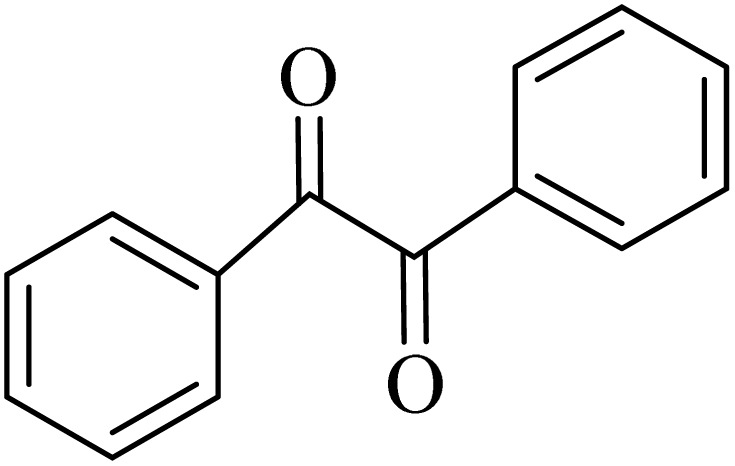	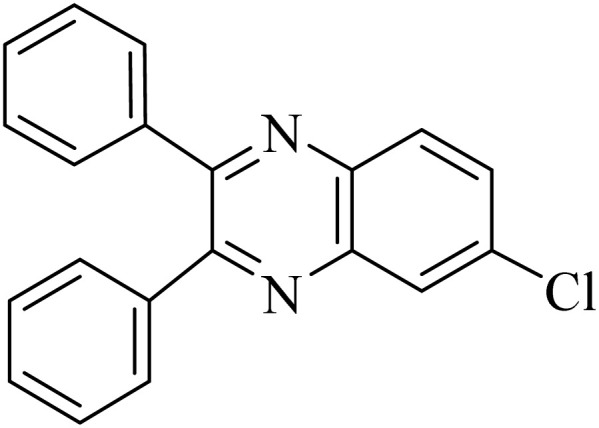	9	92	120–121 (ref. 60)
3	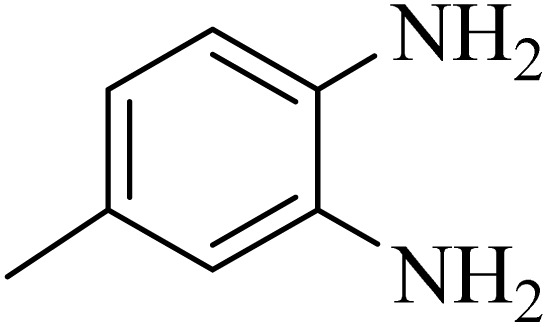	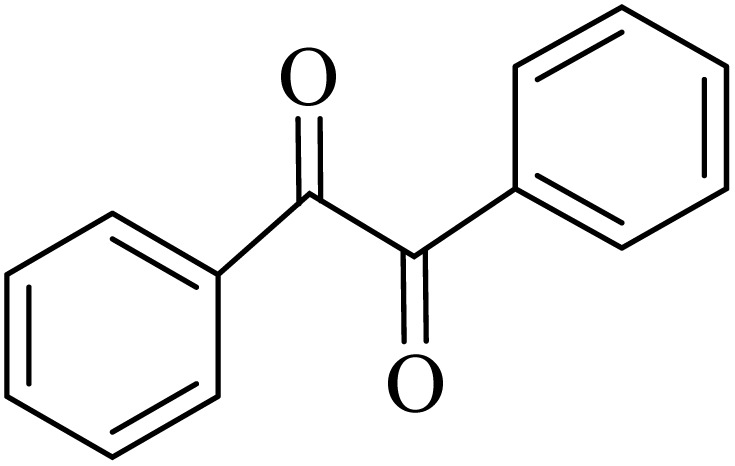	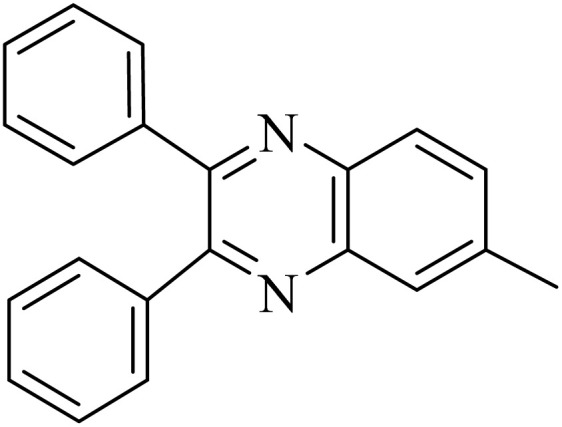	5	92	115–116 (ref. 60)
4	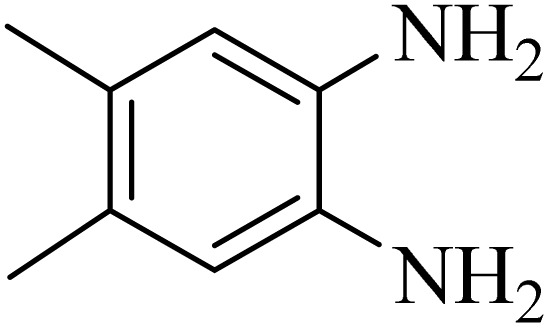	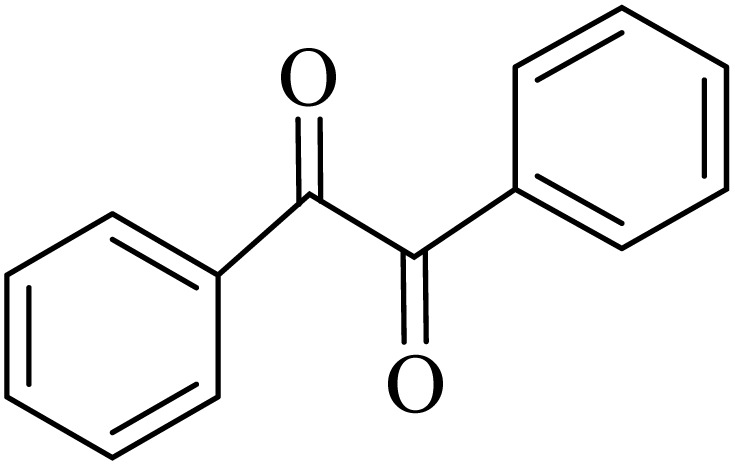	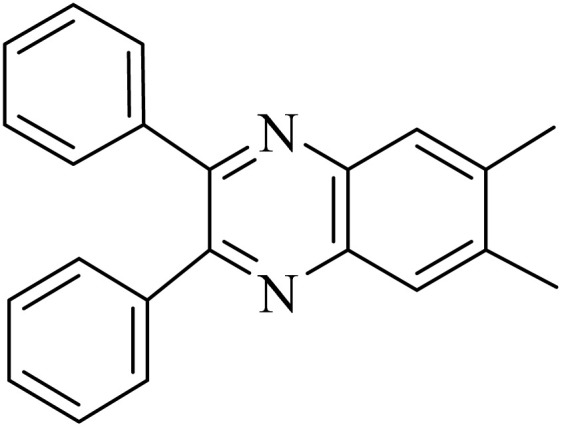	5	92	171–172 (ref. 60)
5	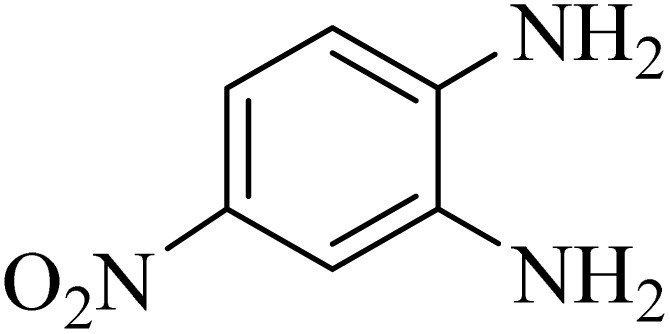	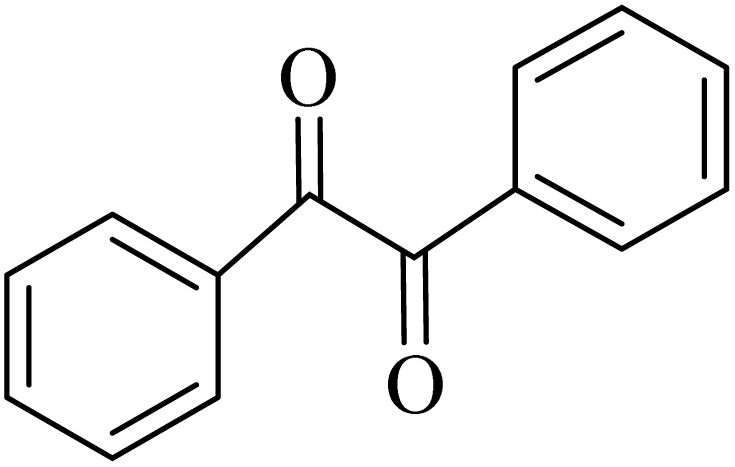	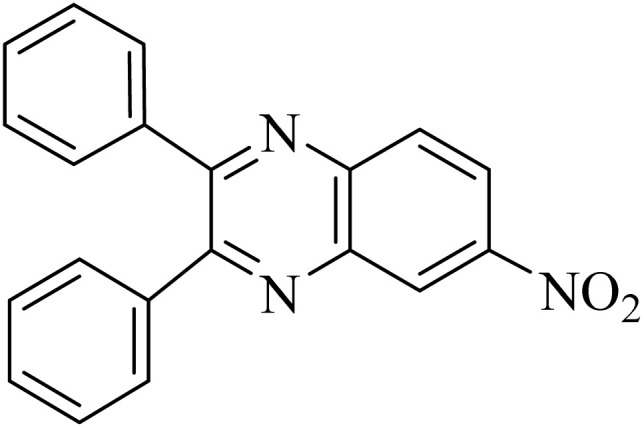	11	80	192–193 (ref. 60)
6	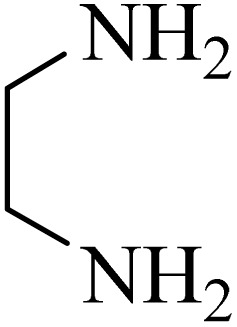	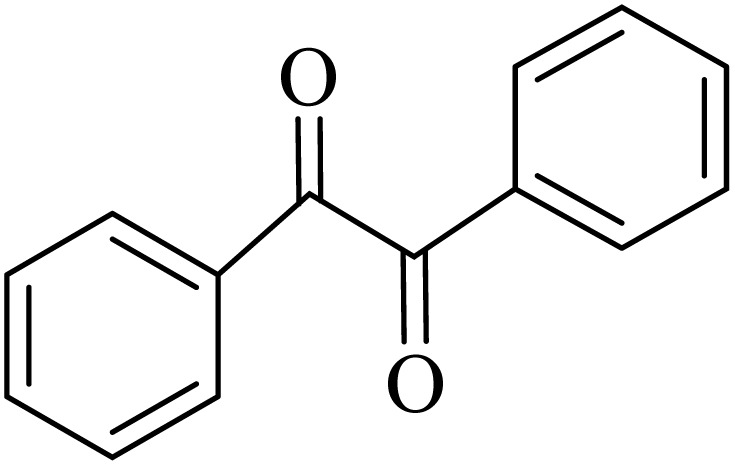	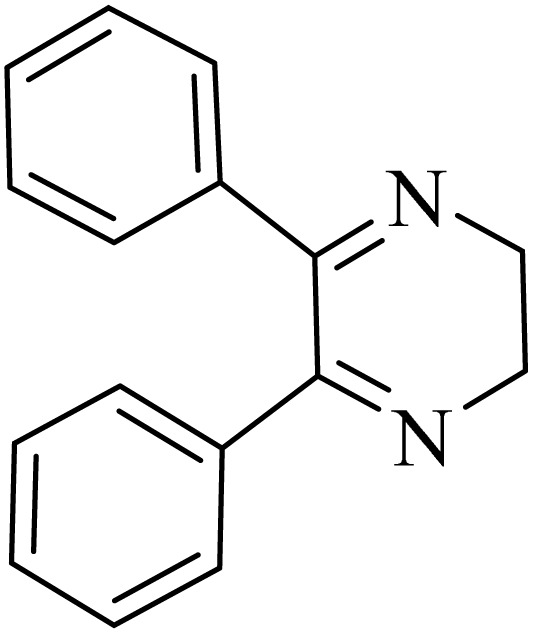	10	89	161–163 (ref. 61)
7	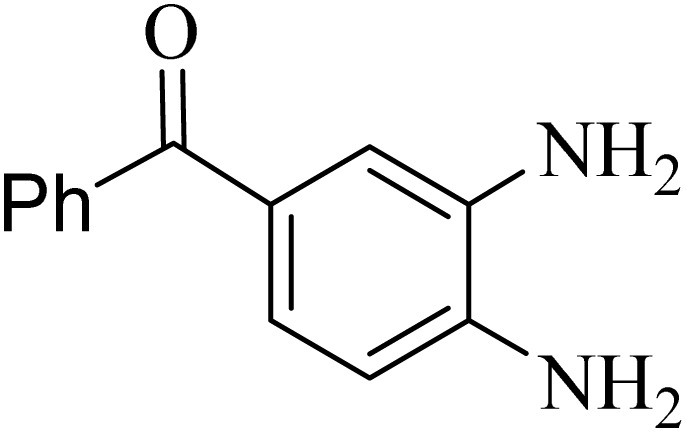	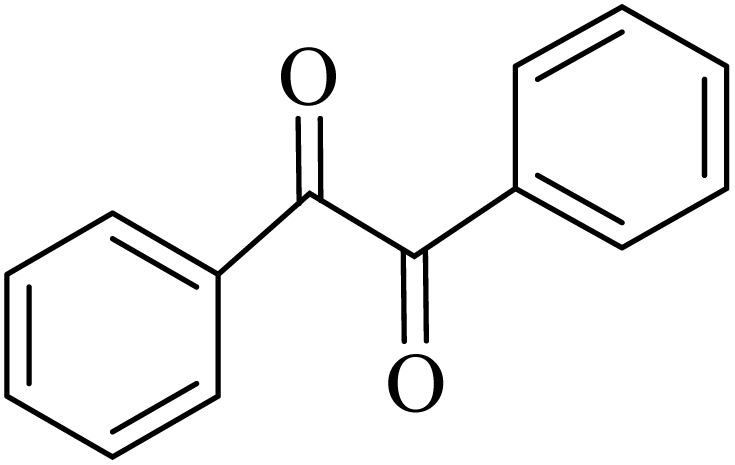	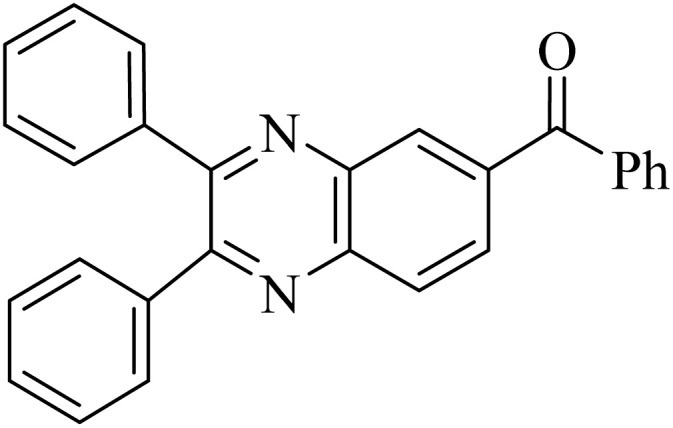	40	88	142–144 (ref. 62)
8	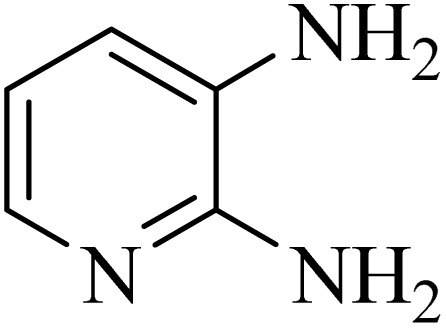	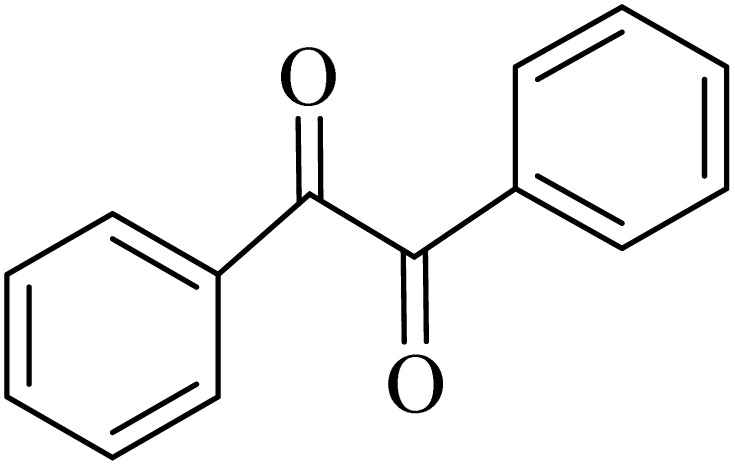	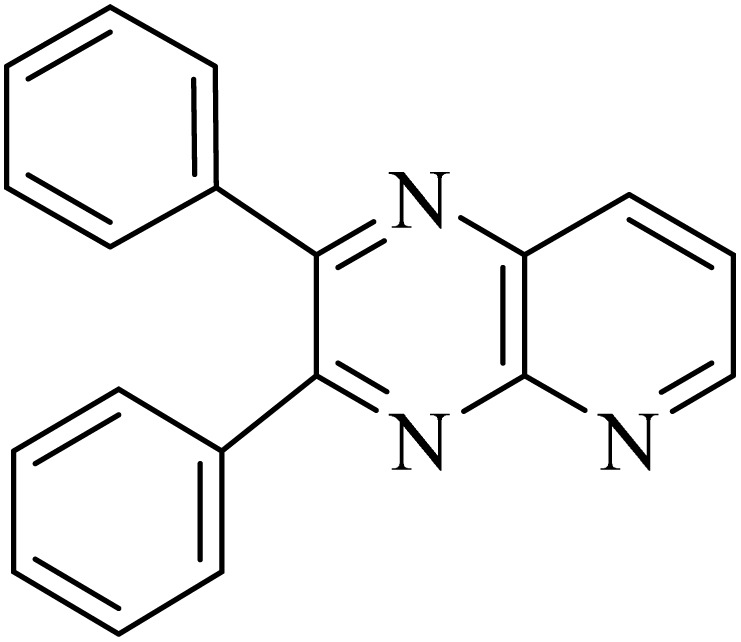	100	60	141–143 (ref. 63)
9	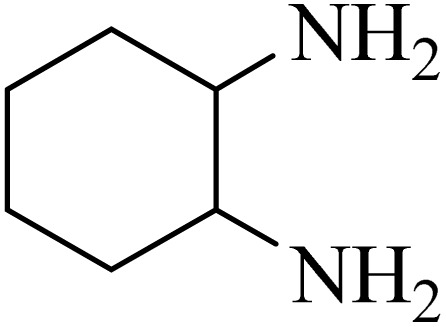	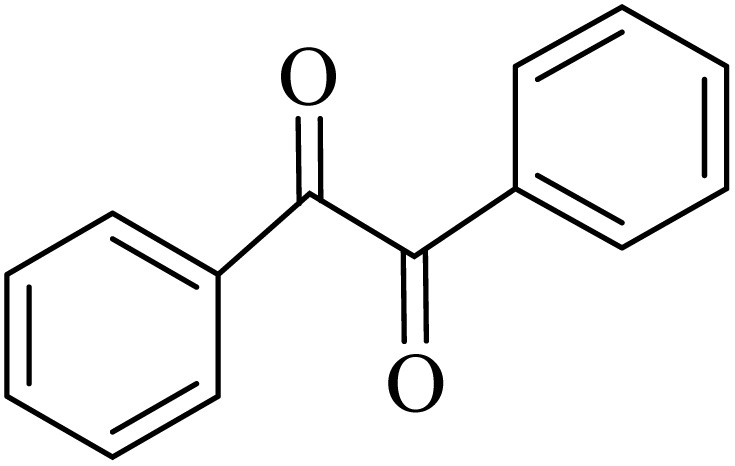	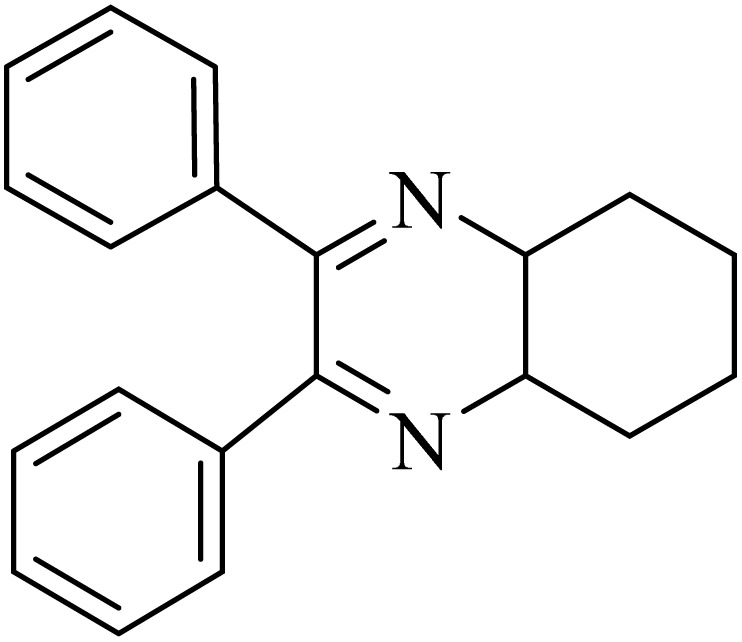	90	70	167–168 (ref. 60)
10	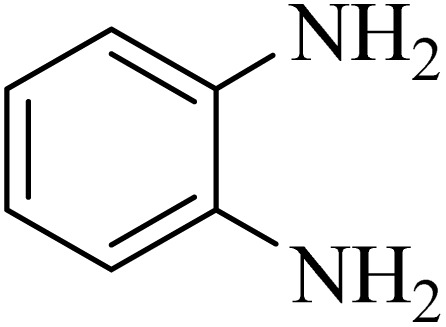	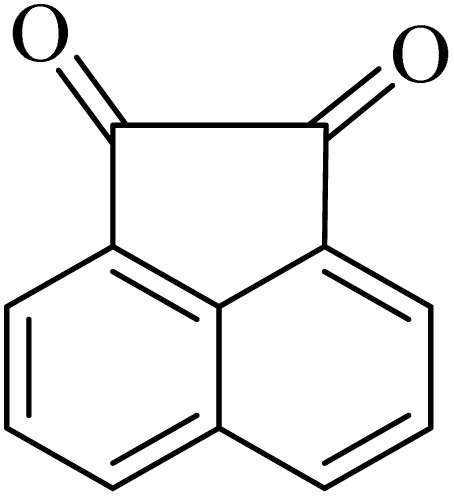	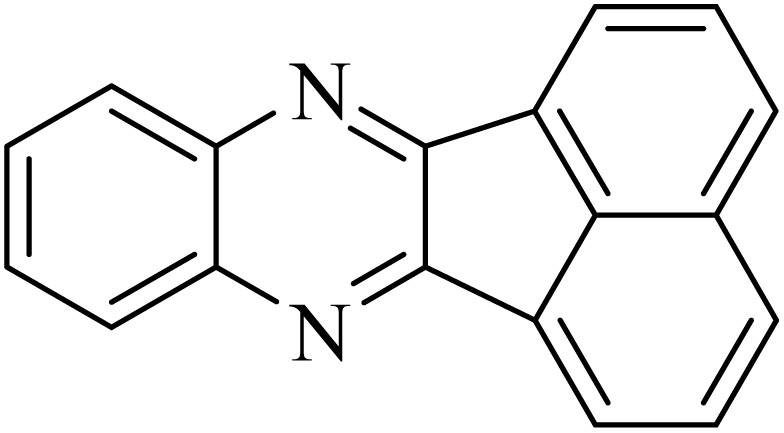	5	98	243–244 (ref. 60)
11	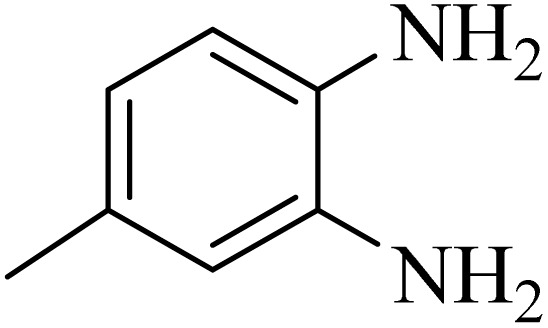	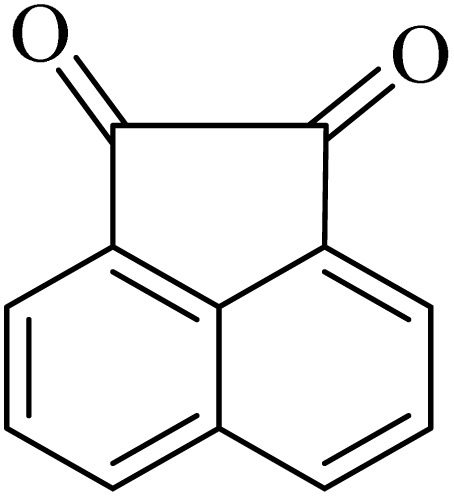	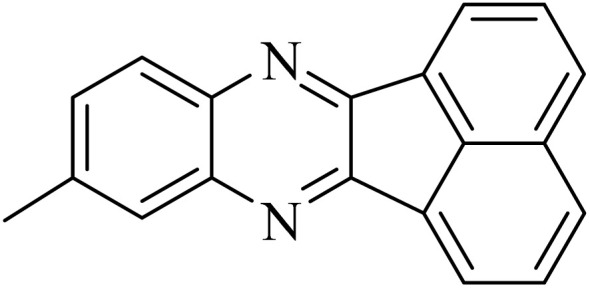	8	98	>300 (ref. 60)
12	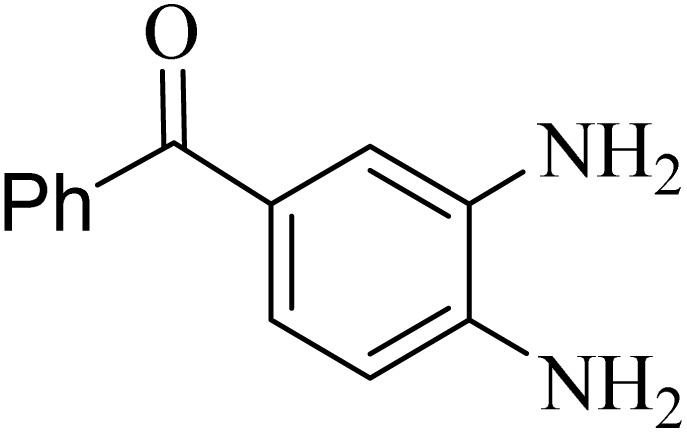	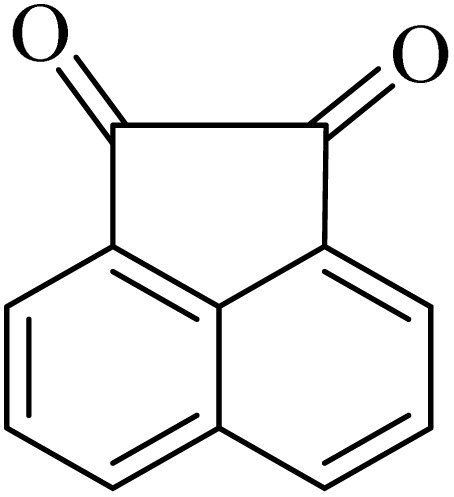	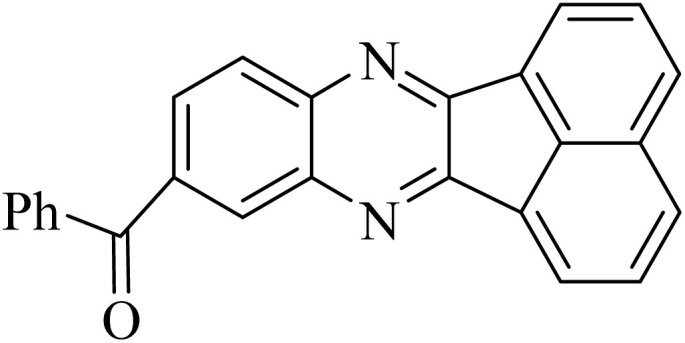	6	98	245–247 (ref. 62)
13	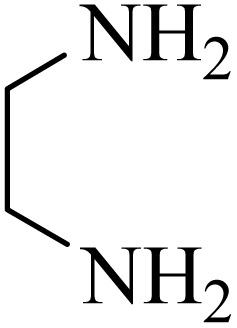	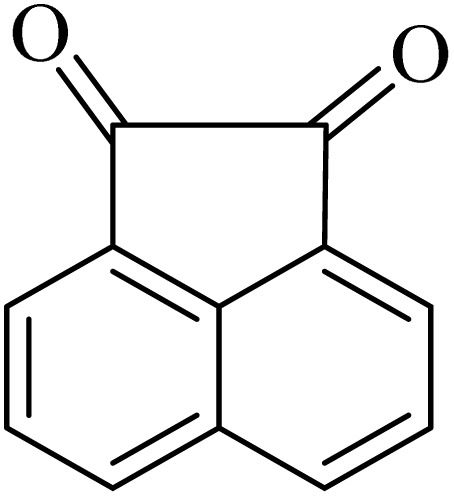	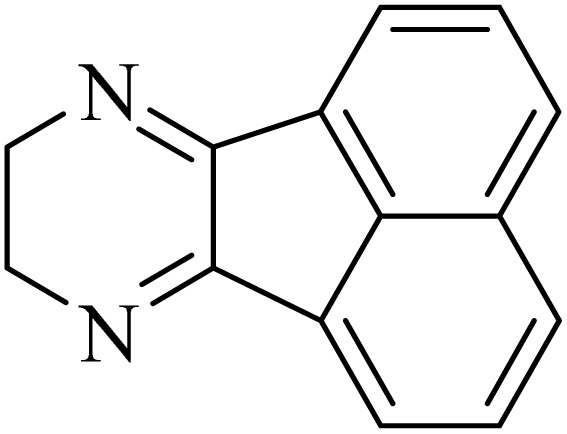	12	97	245–547 (ref. 62)

A comparison between acidic deep eutectic mixture and other catalysts represented in prior publications may help to supplementary survey the catalytic activity of our catalyst. The results are depicted in Table 6, which verifies the benefit of such an approach in preference of the noted procedures, regarding its outstanding efficiency, moderate and eco-friendly conditions, short reaction time, excellent yield, and catalyst recyclability.^64–73^

**Table 6:** Comparative catalytic activity of acidic deep eutectic mixture with other reported catalysts in synthesis of quinoxaline

Entry	Catalyst	Conditions	Time	Yield (%)	Ref.
1	ZrO_2_/Ga_2_O_3_/MCM-41	CH_3_CN, RT	2 h	97	64
2	Graphite	EtOH, RT	1 h	92	65
3	NBS	AcOH, reflux	2.5 h	92	66
4	Nano-g-Fe_2_O_3_–SO_3_H	Solvent-free, 120 °C	1 h	94	67
5	Fe_3_O_4_@SiO_2_@5-SA	EtOH, 60 °C	2–4 h	97	68
6	I_2_, AgNO_3_	MeCN, RT, in the air	4 h	83	69
7	CoBr_2_	DCE, O_2_, 80 °C	24 h	71	70
8	FNHDNi	Toluene-MeOH, 80 °C	2–5 h	96	71
9	ZrO_2_–Al_2_O_3_	DMF, RT	4 h	91	72
10	CeO_2_–ZrO_2_	MeOH, 25 °C	15 min	87	73
**11**	**ADEM**	**EtOH, RT**	**5 min**	**98**	**This work**

### 3.3. Reusability of the acidic deep eutectic mixture

**Fig. 6:**
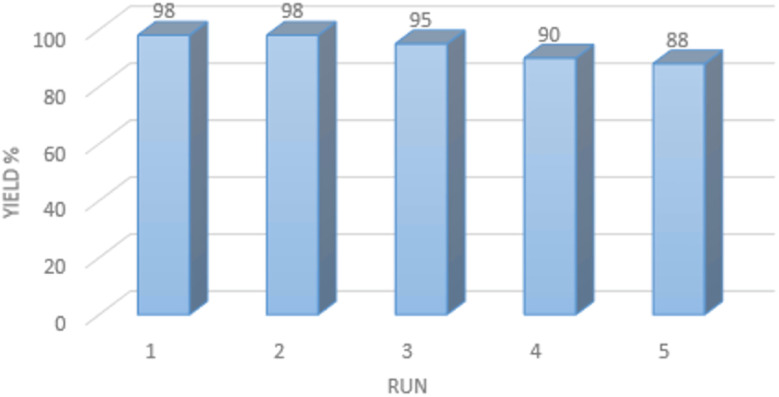
Recyclability of ADEM for the synthesis of quinoxaline.

The final part of this study focuses on assessing the stability of the prepared acidic deep eutectic mixture catalyst by evaluating its reusability in the direct synthesis of quinoxaline, as depicted in Fig. 6. To achieve this objective, model reactions were conducted on a 10 mmol scale in the presence of 1 mL of ADEM. Upon completion of the reaction, 20 mL of ethyl acetate was added, leading to phase separation. The ethyl acetate phase was removed, and the ADEM phase was retained for subsequent runs. Even after five cycles of recycling, no significant decrease in either the activity or efficiency of the urea/SbCl_3_/HCl catalyst was observed, demonstrating its reusability. Furthermore, analysis of the reused catalyst using FTIR spectroscopy revealed no substantial changes compared to the freshly prepared system (Fig. S1 in ESI†).

## 4. Conclusions

In summary, ADEM was synthesized for the first time *via* a facile and economical approach employing an inexpensive and available substrate. The introduced ADEM catalyst plays a pivotal role in the synthesis of quinazolinone and quinoxaline derivatives, effectively addressing concerns related to operational simplicity and cost-efficiency. The existing system proved vital to ensure efficient conversion since the product yield was too scant in the absence of such a medium for a characteristic reaction, while the time was also protracted. This protocol bears several merits, including time-saving reaction, mild conditions, notable purity of products, excellent yields, and reusability of the system. Moreover, the reusability of the catalyst was explored in five successive runs, showing no substantial drop in the catalyst activity.

## Author contributions

Fatemeh Mohammad: methodology, investigation, data curation, writing – original draft. Najmedin Azizi: conceptualization, methodology and writing – review & editing. Zohreh Mirjafari: supervision and writing – original Draft. Javad Mokhtari: formal analysis, investigation. All authors approved the final approval of version to be published and agreed to be accountable for all aspects of the work in ensuring that questions related to the accuracy or integrity of any part of the work are appropriately investigated and resolved.

## Conflicts of interest

Authors declare no conflicts of interest.

## Supplementary Material

RA-015-D5RA03346B-s001

## Data Availability

The data that support the findings of this study are available from the corresponding author, [Najmedin Azizi], upon reasonable request.
