# Supplementary material for: Catalytic acidic deep eutectic mixture for efficient and promising synthesis of quinazolinone and quinoxaline derivatives
Source: RSC Adv. 2025 Jul 21;15(32):25971–84. doi: 10.1039/d5ra03346b (PMC12279010; doi:10.1039/d5ra03346b)
Supplement: RA-015-D5RA03346B-s001 [file RA-015-D5RA03346B-s001.pdf]

Supplementary Information (SI) for RSC Advances.
This journal is © The Royal Society of Chemistry 2025

Supplementary File

Catalytic acidic deep eutectic mixture for Efficient and promising synthesis of quinazolinone and quinoxaline derivatives

Fatemeh Mohammad[a], Najmedin Azizi[b*], Zohreh Mirjafari[a], Javad Mokhtari[a]

[a] Department of Chemistry, Science and Research Branch, Islamic Azad University, Tehran, Iran

[b] Chemistry and Chemical Engineering Research Center of Iran, P.O. Box 14335-186, Tehran, Iran.

*Corresponding authors: azizi@ccerci.ac.ir

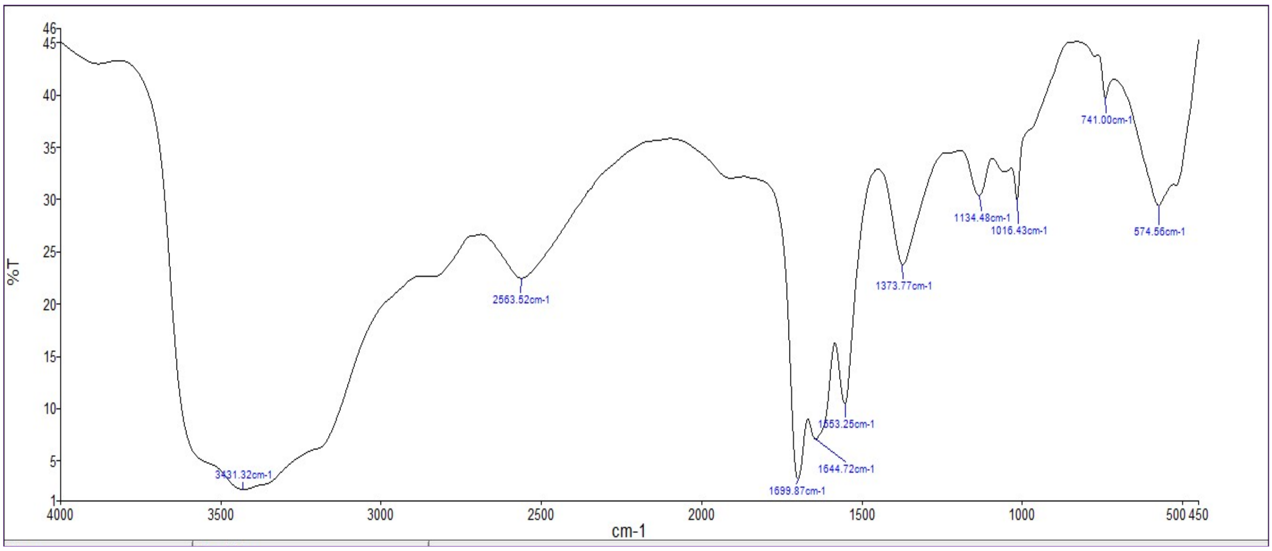

Fig. S1: FT IR of reused **acidic deep eutectic mixture**

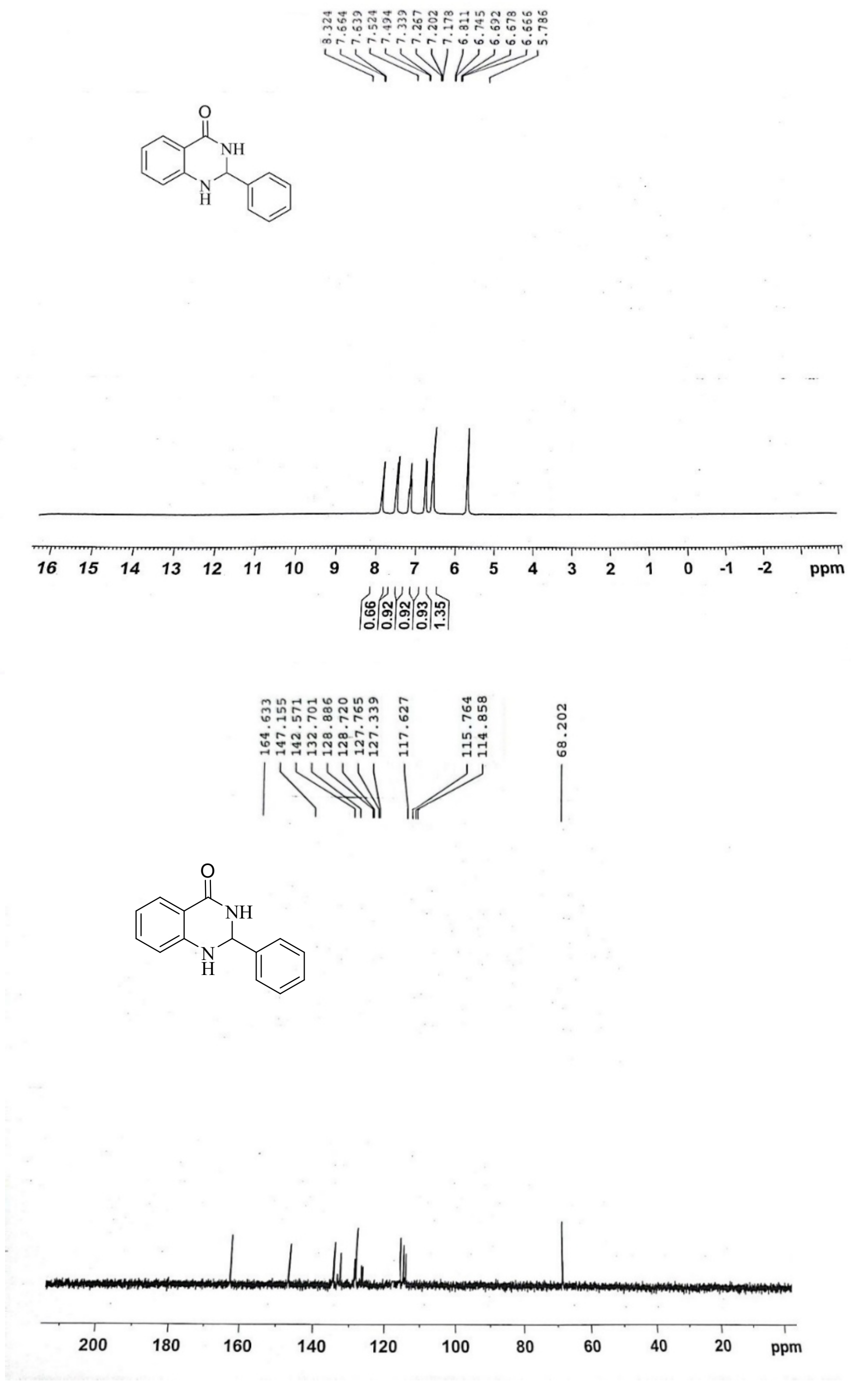

Fig. S2: ^{1}H and ^{13}C NMR of 2-Phenyl-2,3-dihydroquinazolin-4(1H)-one

2-Phenyl-2,3-dihydroquinazolin-4(1H)-one :

^{1}HNMR (500 MHz, DMSO-d6): 5.78 (s, 1H), 6.66-6.69 (m, 1H), 6.74-6.81 (d, J 8.02 Hz, 1H), 7.17 (s, 1H), 7.20-7.26 (m, 1H), 7.33-7.49 (m, 3H), 7.52-7.63 (d, J= 7.30 Hz, 2H), 7.66 (s, J 7.30 Hz, 1H), 8.32 (s, 1H); ^{13}C NMR (125 MHz, DMSO-d6): 67.03, 114.86, 115.43, 117.56, 127.30, 127.81, 128.77, 128.89, 133.75, 142.13, 148.31, and 164.03.

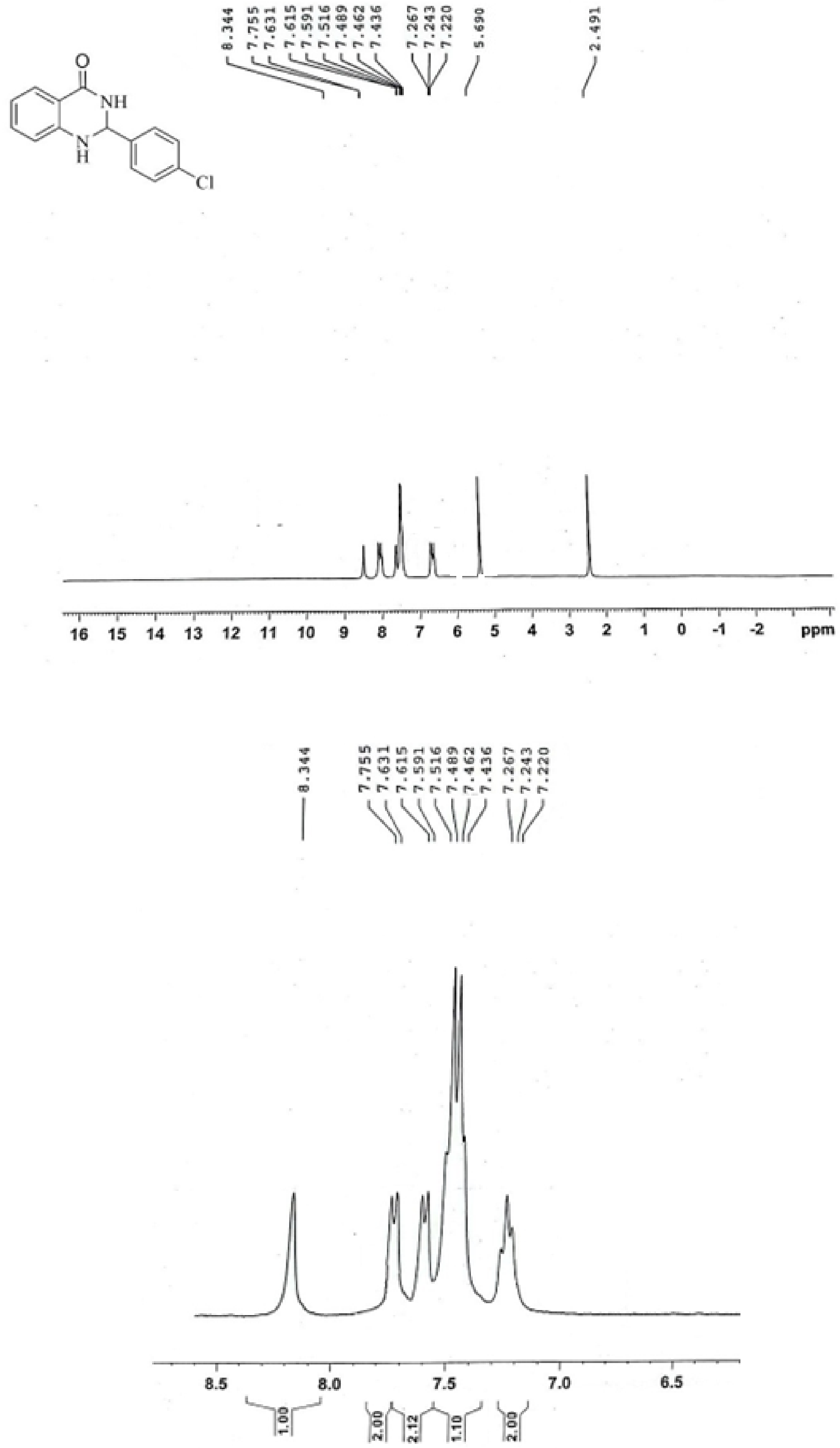

Fig. S3: ^{1}H NMR of 3-(4-chlorophenyl)-2-methylquinazolin-4(3H)-one

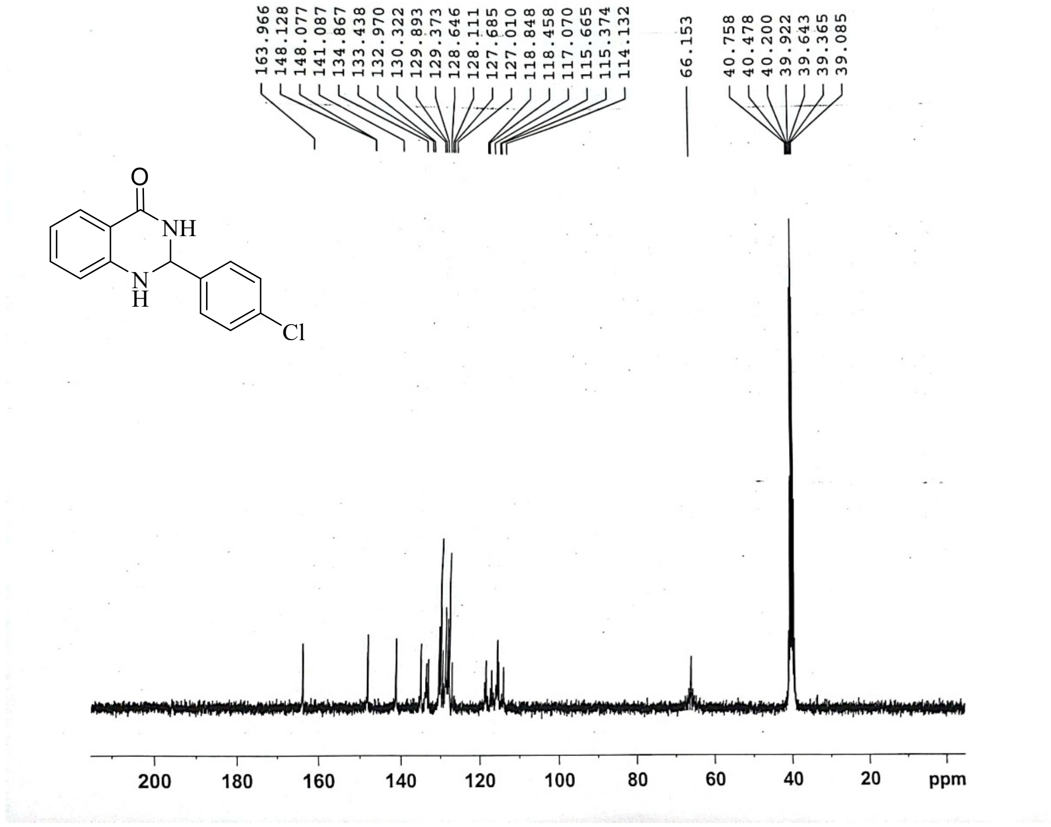

Fig. S4: ^{13}C NMR of 3-(4-chlorophenyl)-2-methylquinazolin-4(3H)-one

3-(4-chlorophenyl)-2-methylquinazolin-4(3H)-one :

^{1}H NMR (DMSO-d6, 500 MHz): 2.49 (s, 3H, CH3), 5.96 (s, 1H), 7.22–7.26 (m, 2H, ArH), 7.43–7.51 (m, 1H, ArH), 7.59 (d, J= 6.90 Hz, 2H, ArH), 7.61–7.75 (m, 2H, ArH), 8.34 (t, J= 8.1 Hz, 1H, ArH) ppm. ^{13}C NMR (125 MHz, DMSO-d6): d 66.15, 120.6, 126.8, 127.0, 129.5, 130.1, 130.3, 134.4, 135.4, 136.2, 147.4, 153.7, 162.1 .

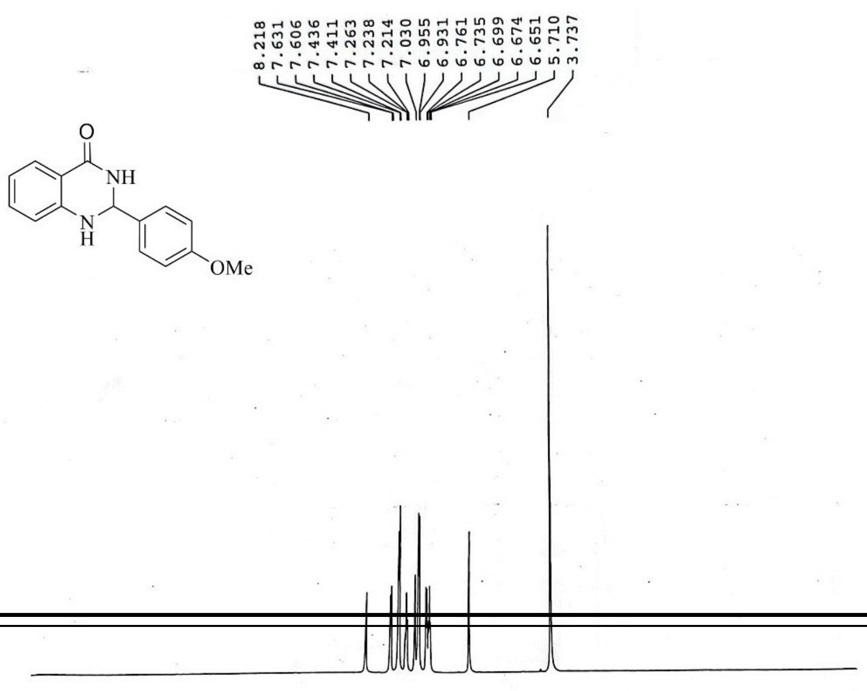

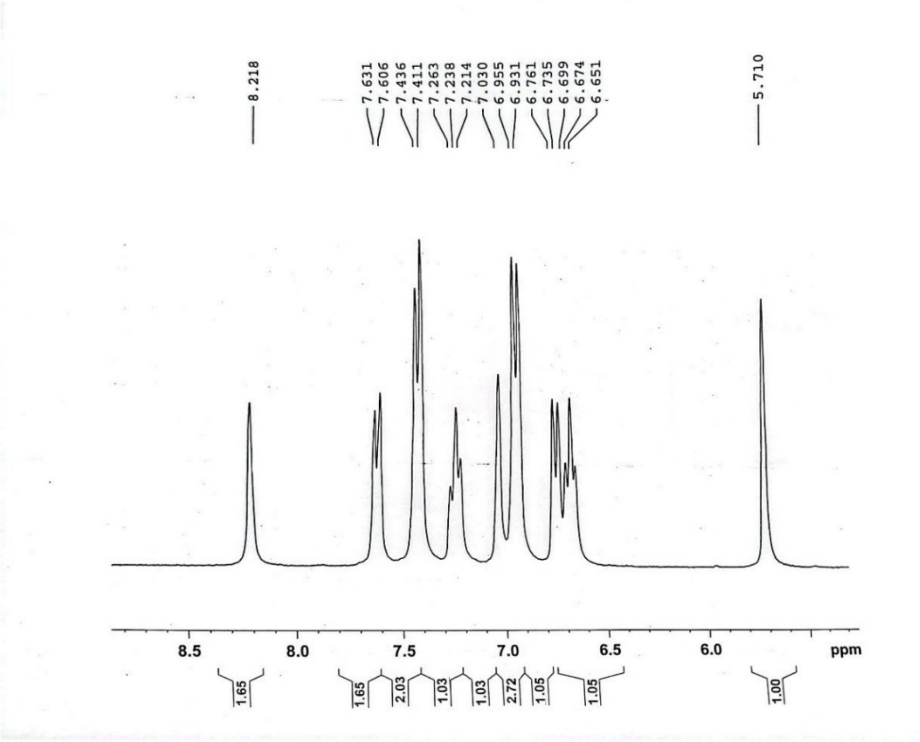

Fig. S5: ^{1}H NMR of 2-(4-Methoxyphenyl)-2,3-dihydroquinazolin-4(1H)-one

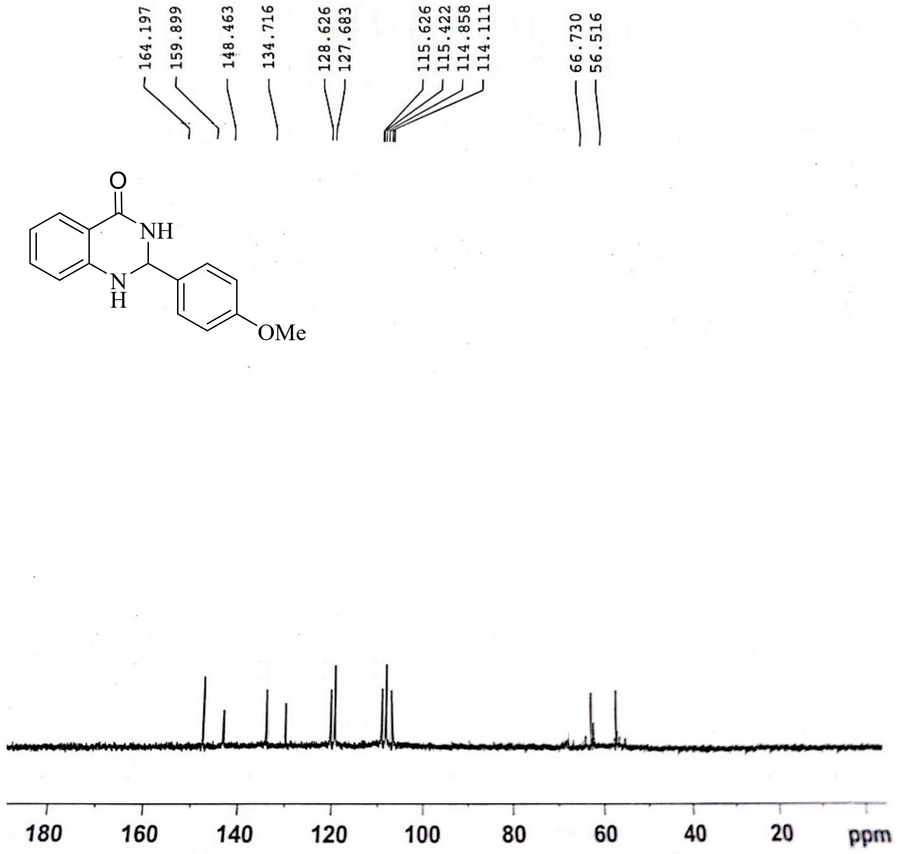

Fig. S6: ^{13}C NMR of 2-(4-Methoxyphenyl)-2,3-dihydroquinazolin-4(1H)-one

2-(4-Methoxyphenyl)-2,3-dihydroquinazolin-4(1H)-one :

^{1}HNMR (500 MHz, DMSO-d6): 3.73 (s, 3H), 5.71 (s, 1H), 6.65-6.69 (t, J = 7.39 Hz, 1H), 6.73-6.76 (d, J= 8.08 Hz, 1H), 6.93-6.95 (d, J =8.62 Hz, 2H), 7.03 (s, 1H), 7.21-7.26 (t, J =7.60 Hz, 1H), 7.41-7.43 (d, J= 8.62 Hz, 2H), 7.60-7.63 (d, J =7.67 Hz, 1H), 8.21 (s, 1H); ^{13}C NMR (125 MHz, DMSO-d6): 55.51, 66.73, 114.11, 114.85, 115.42, 115.62, 127.68, 128.62, 134.71, 148.46, 159.89, 164.19.

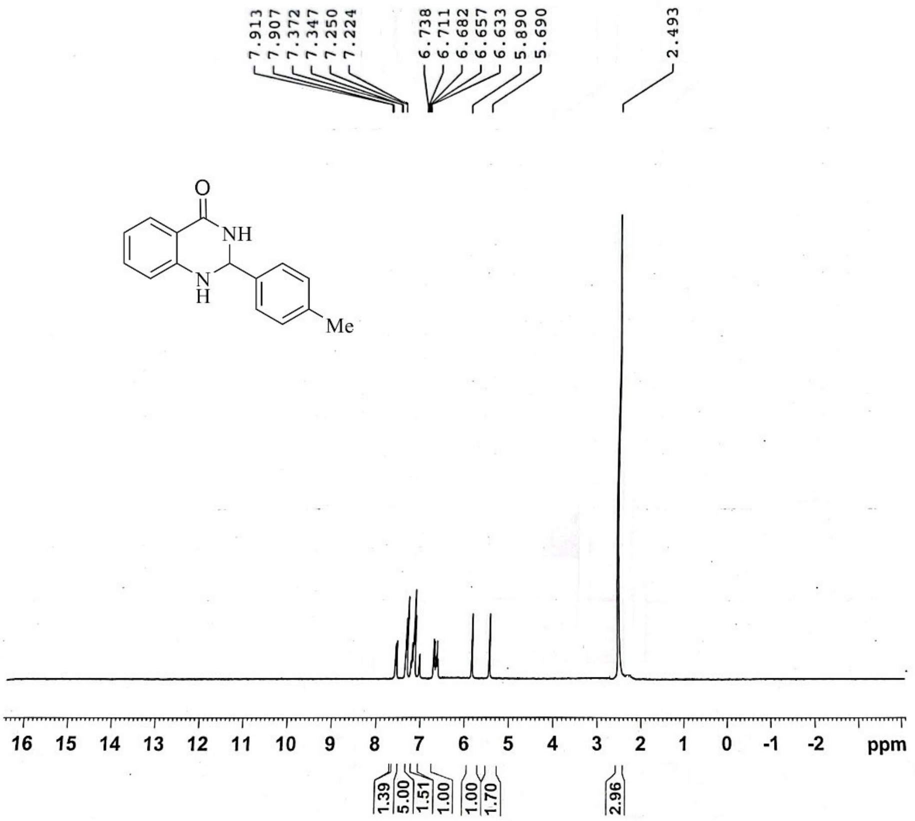
7.913
7.907
7.372
7.347
7.250
7.224
6.738
6.711
6.682
6.657
6.633
5.890
5.690
2.493
O
NH
N
H
Me
16 15 14 13 12 11 10 9 8 7 6 5 4 3 2 1 0 -1 -2 ppm
1.39
5.00
1.51
1.00
1.00
1.70
2.96

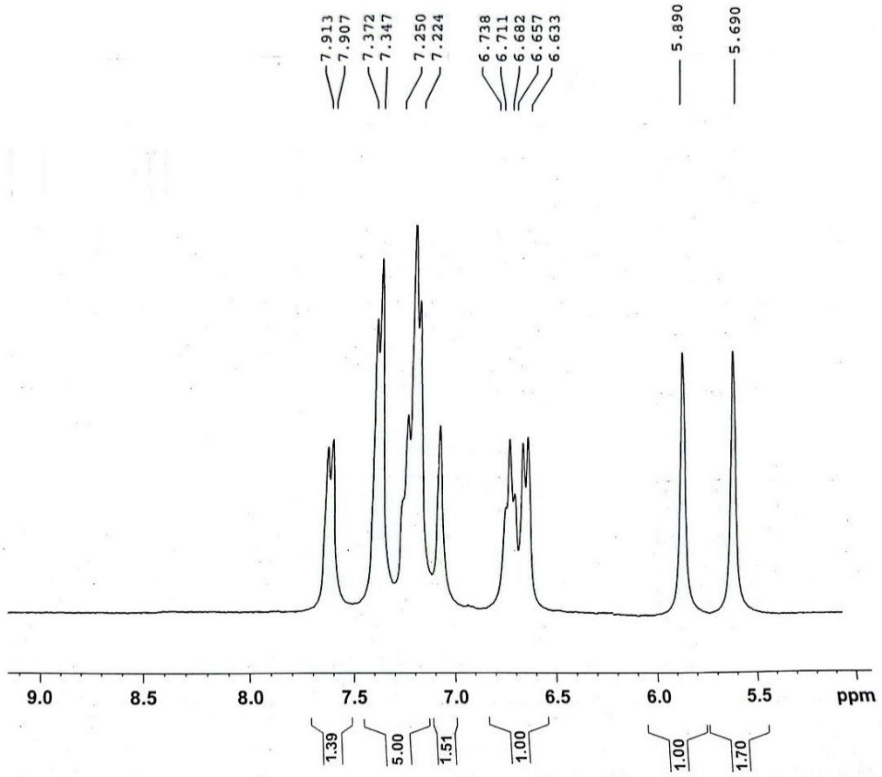
7.913
7.907
7.372
7.347
7.250
7.224
6.738
6.711
6.682
6.657
6.633
5.890
5.690
9.0 8.5 8.0 7.5 7.0 6.5 6.0 5.5 ppm
1.39
5.00
1.51
1.00
1.00
1.70

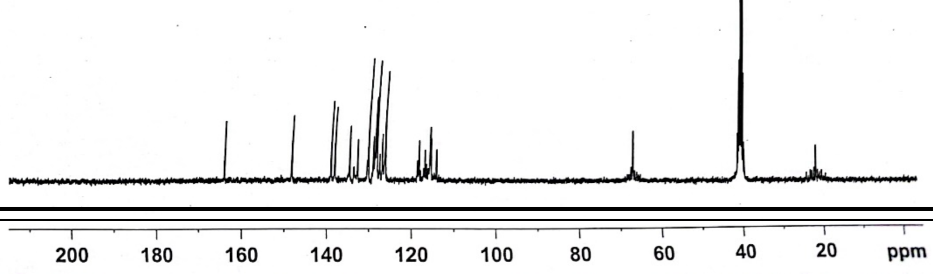
200 180 160 140 120 100 80 60 40 20 ppm

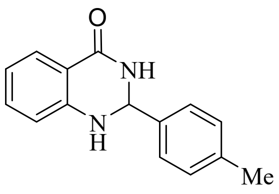

Fig. S7: ^{1}H NMR and ^{13}C NMR of 2-(p-tolyl)-2,3-dihydroquinazolin-4(1H)-one

2-(p-tolyl)-2,3-dihydroquinazolin-4(1H)-one :

^{1}H NMR (500 MHz, DMSO-d6): 2.49 (s, 3H), 5.69 (s, 1H), 5.89 (s, 1H), 6.63-6.65 (d, 1H, J= 8.1Hz), 6.68-6.73 (t, 1H, J= 7.5Hz), 7.22-7.37 (m, 5H), 7.90-7.91 (d, 1H, J= 7.8Hz) ; ^{13}C NMR (125 MHz, DMSO-d6): δ 21.4, 67.10, 114.8, 117.5, 124.4, 127.7, 127.9, 128.6, 129.5, 133.7, 137.8,141.9, 148.3, 154.1, 164.1.

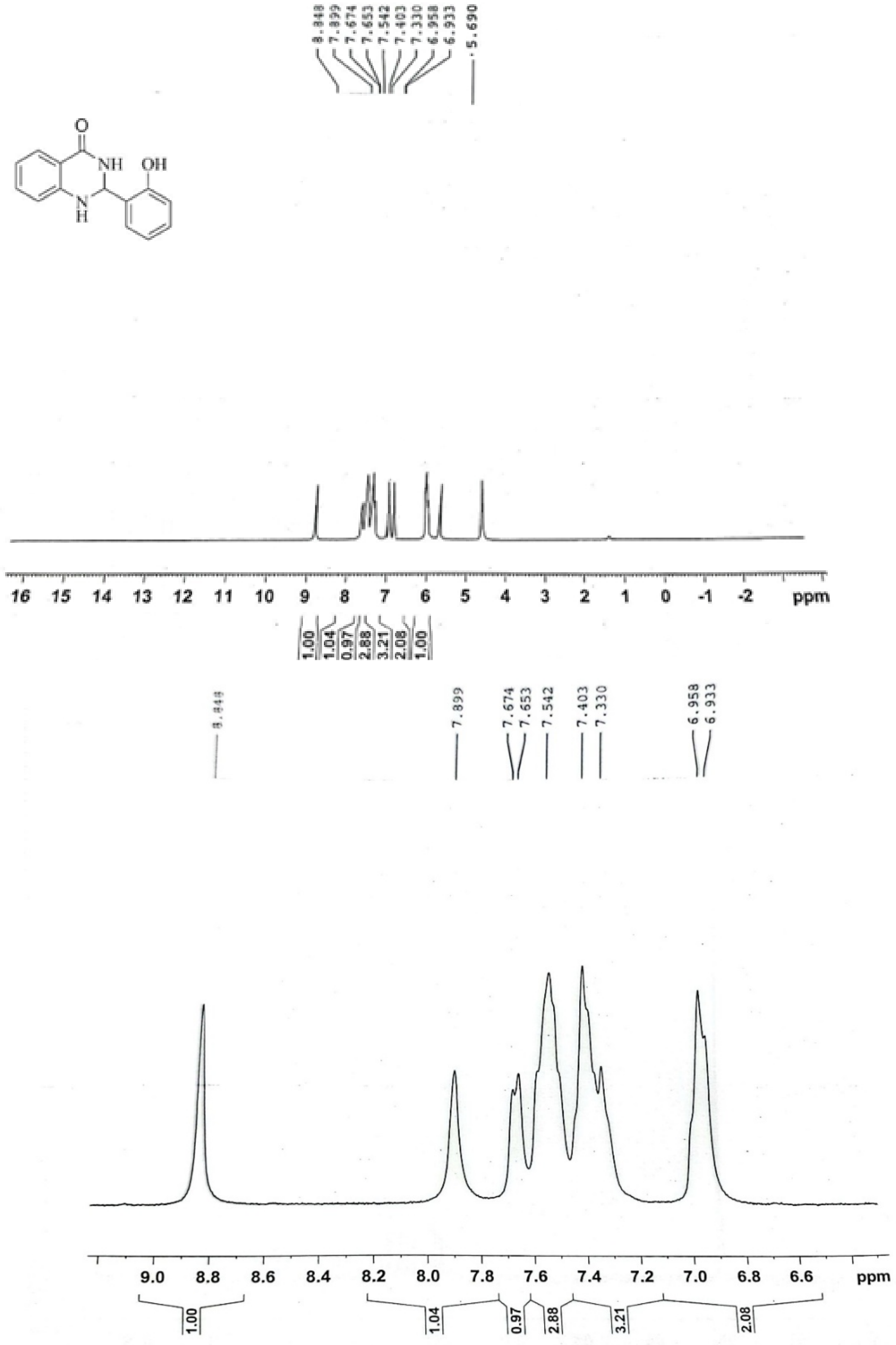

Fig. S8: ^{1}H NMR and ^{13}C NMR of 2-(2-hydroxyphenyl)-2,3-dihydroquinazolin-4(1H)-one

2-(2-hydroxyphenyl)-2,3-dihydroquinazolin-4(1H)-one :

^{1}H NMR (500 MHz, DMSO-d6): δ 4.61 (s, 1H), 5.89 (s, 1H), 5.92 (s, 1H), 6.59 (m, 4H), 6.70 (d, 1H, J=7.8 Hz), 7.10 (d, 1H, J=7.5 Hz), 7.11 (m, 2H), 8.95 (s, 1H); ^{13}C NMR (125 MHz, DMSO-d6): δ 70.9, 129.4, 128.4, 129.6, 129.8, 132.4, 132.9, 133.7, 135.6, 141.5, 155.7, 165.6.

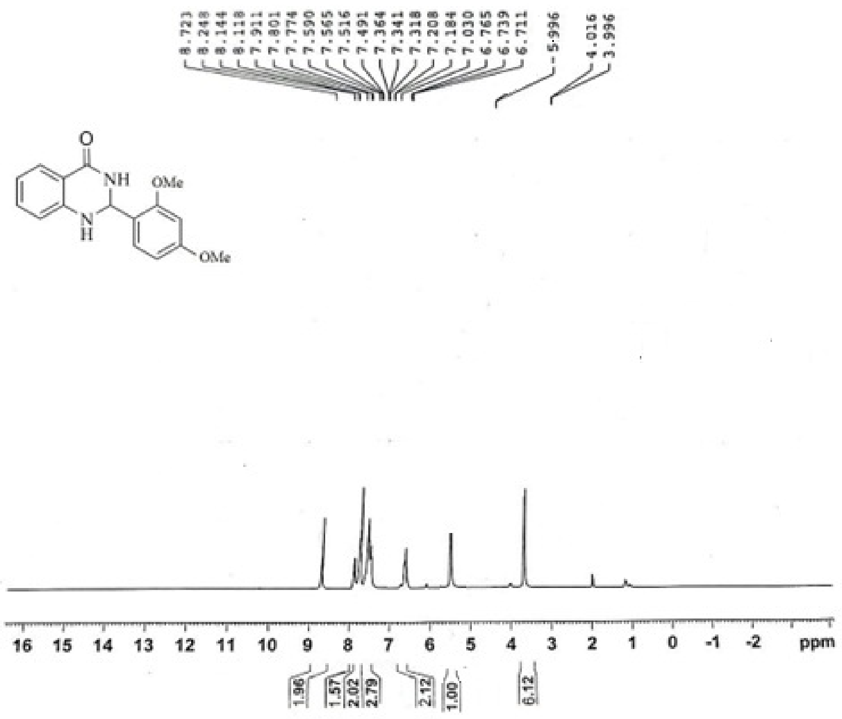
8.723 8.248 8.144 8.118 7.911 7.801 7.774 7.590 7.565 7.516 7.491 7.364 7.341 7.318 7.208 7.184 7.030 6.765 6.739 6.711
5.996
4.016 3.996
O
NH
OMe
N H
OMe
16 15 14 13 12 11 10 9 8 7 6 5 4 3 2 1 0 -1 -2 ppm
1.96 1.57 2.02 2.79 2.12 1.00 6.12

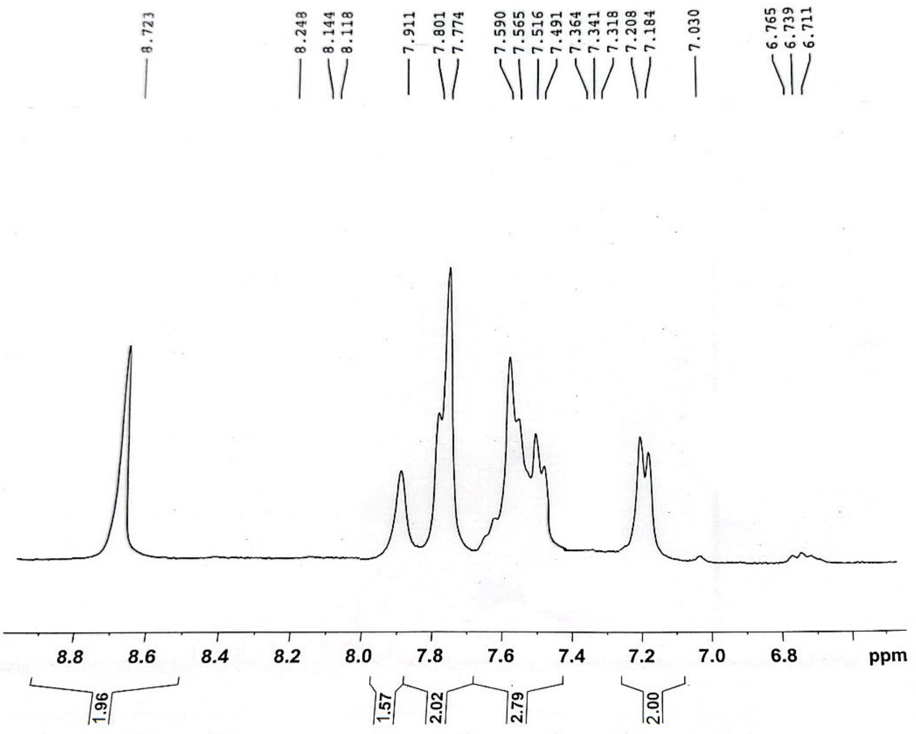
8.723
8.248 8.144 8.118
7.911 7.801 7.774
7.590 7.565 7.516 7.491 7.364 7.341 7.318 7.208 7.184
7.030
6.765 6.739 6.711
8.8 8.6 8.4 8.2 8.0 7.8 7.6 7.4 7.2 7.0 6.8 ppm
1.96 1.57 2.02 2.79 2.00

Fig. S9: ^{1}H NMR of 2-(2,4-dimethoxyphenyl)-2,3-dihydroquinazolin-4(1H)-one

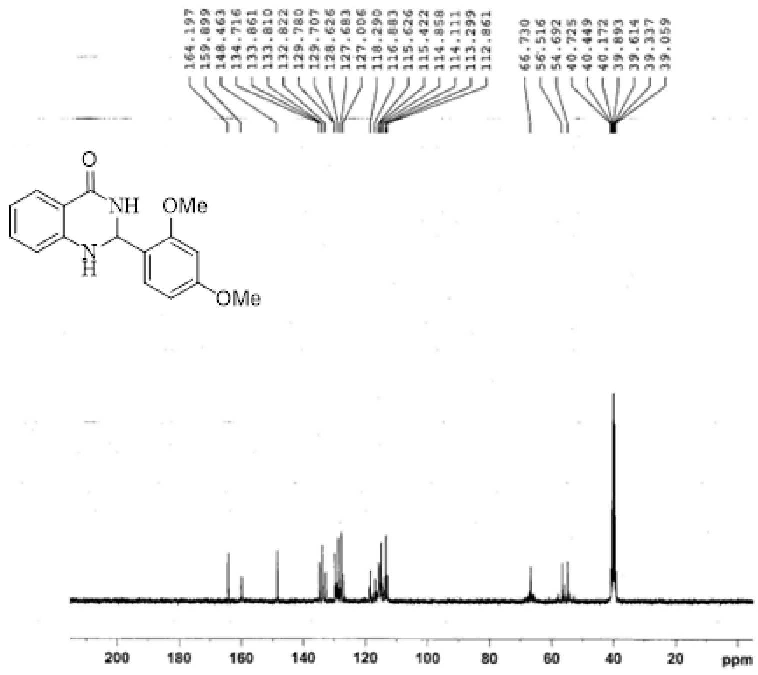

Fig. S10: ^{13}C NMR of **2-(2,4-dimethoxyphenyl)-2,3-dihydroquinazolin-4(1H)-one**

2-(2,4-dimethoxyphenyl)-2,3-dihydroquinazolin-4(1H)-one :

^{1}H NMR (500 MHz, DMSO-d6): 3.85 (s, 6H), 6.68 (t, 2H), 7.71 (m, 2H), 7.99 (d, 2H, J= 8.1Hz), 8.14 (d, 1H, J= 7.5Hz), 7.90-7.91 (d, 1H, J= 7.8Hz) 9.63 (s, 1H), ; ^{13}C NMR (125 MHz, DMSO-d6):): δ 21.4, 55.85, 56.20, 99.71, 105.34, 122.01, 122.7, 128.01, 128.1, 133.1, 134.9,150.9, 159.3, 160.1, 163.1, 164.1.

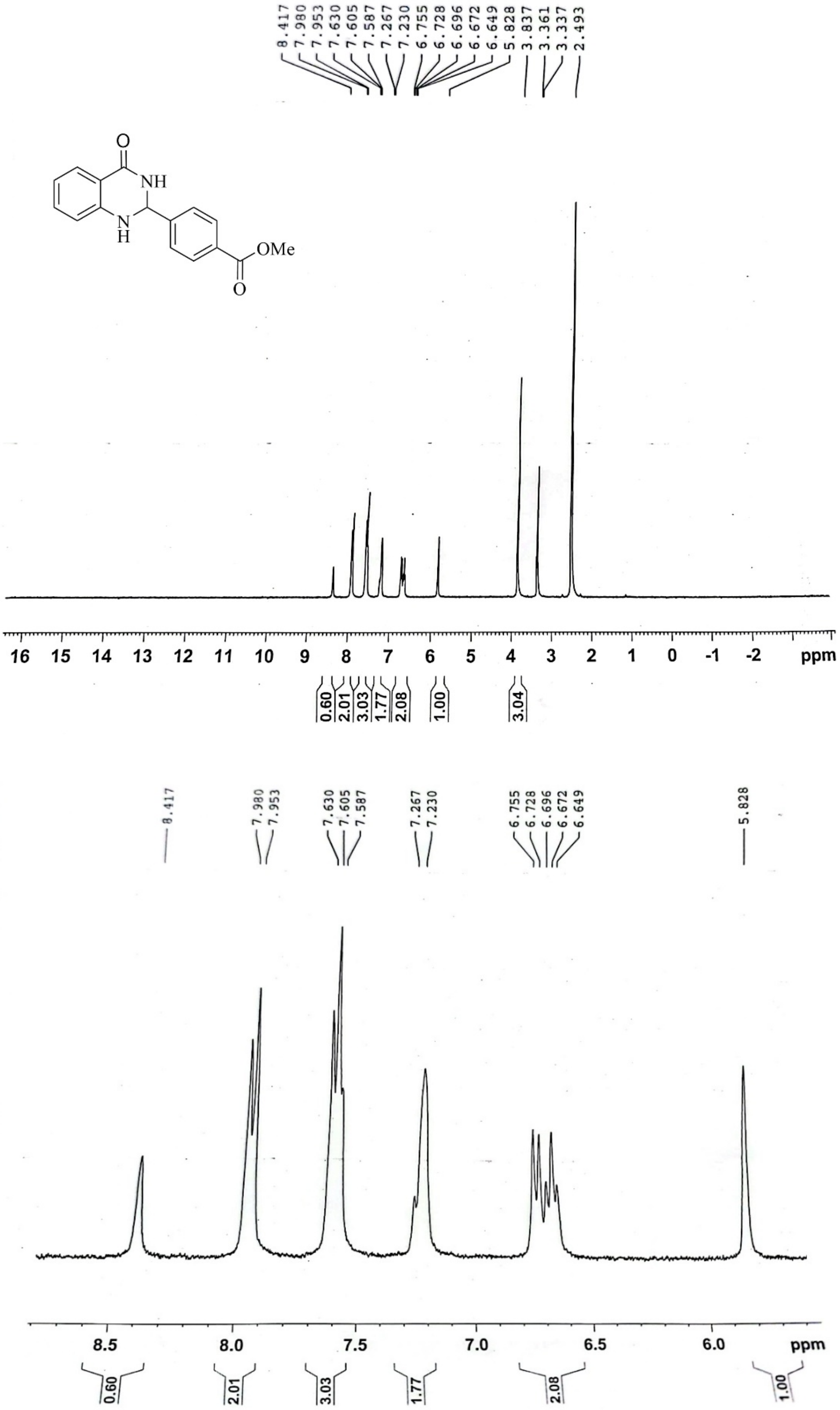

Fig. S11: ^{1}HNMR of 2-(4-methylformylephenyl)-2,3-dihydroquinazolin-4(1H)-one

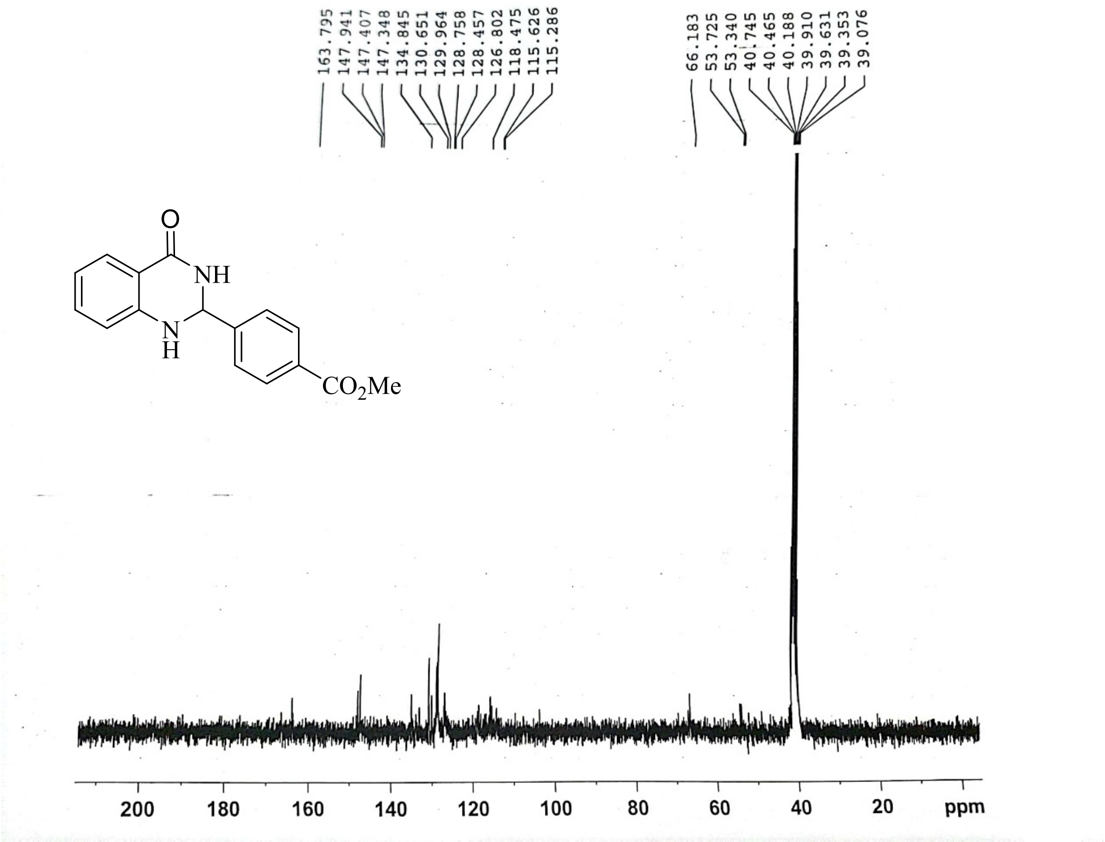

Fig. S12: ^{1}HNMR of 2-(4-methyl formylephenyl)-2,3-dihydroquinazolin-4(1H)-one

2-(4-methyl formylephenyl)-2,3-dihydroquinazolin-4(1H)-one:

^{1}HNMR (500 MHz, DMSO-d6): 3.83 (s, 3H), 5.83 (s, 1H), 6.65 (s, 1H), 6.66-7.95 (m, 8H), 8.43 (s, 1H),); 13C NMR (125 MHz, DMSO-d6): 54.03, 66.86, 115.43, 115.50, 118.56, 126.30, 128.10, 129.81, 130.77, 147.49, 147.91, 163.13, 165.31.

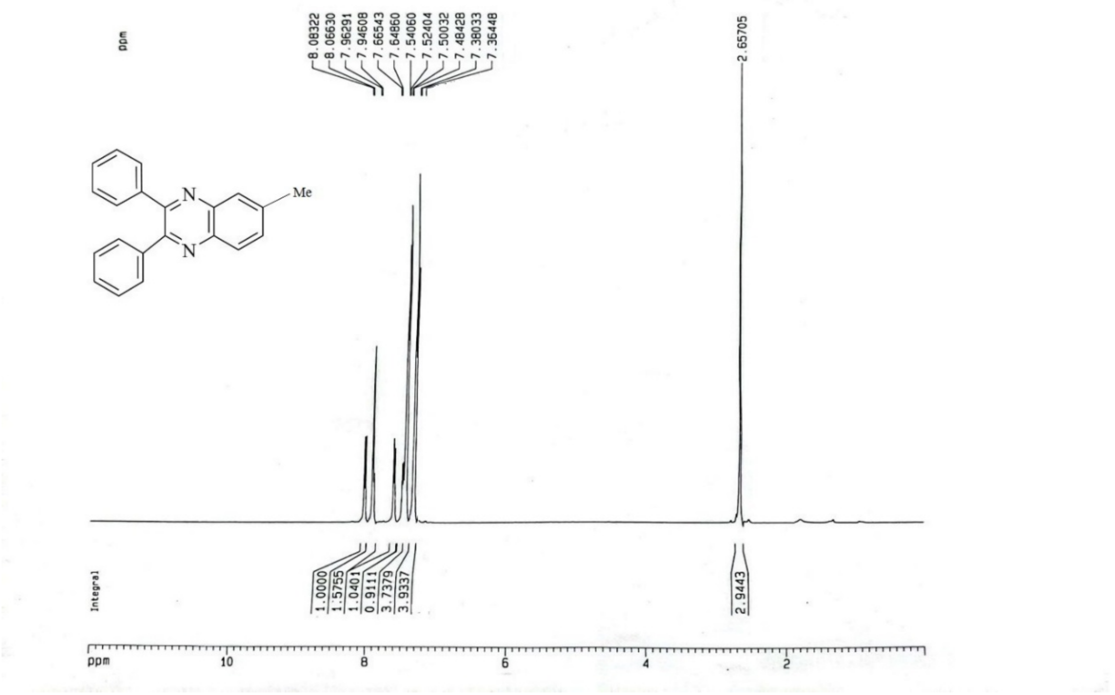

Fig. S13: ^{1}HNMR of 6-methyl-2,3-diphenylquinoxaline

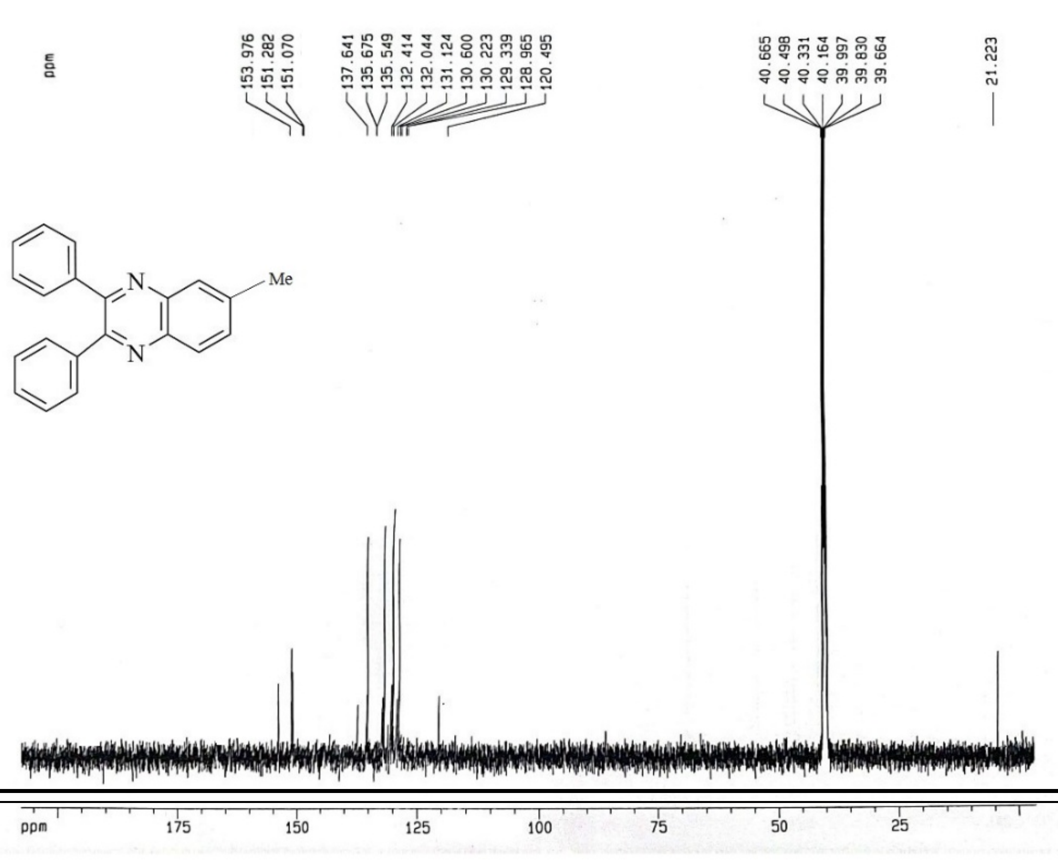

Fig. S14: ^{13}CNMR of 6-methyl-2,3-diphenylquinoxaline

^{1}HNMR (500 MHz, DMSO-d6): 2.53 (s, 3H), 7.30 (m, 1H), 7.65 (m, 1H), 6.74 (m, 2H), 8.03 (d, 1H),), 8.11 (s, 1H), 9.21 (s, 1H); ^{13}C NMR (125 MHz, DMSO-d6): 21.03, 76.86, 77.03, 77.30, 124.56, 128.30, 128.90, 128.99, 129.32, 129.49, 130.11, 130.87, 130.60, 130.87, 35.58, 138.78 141.39, 142.13, 143.37, 153.89.

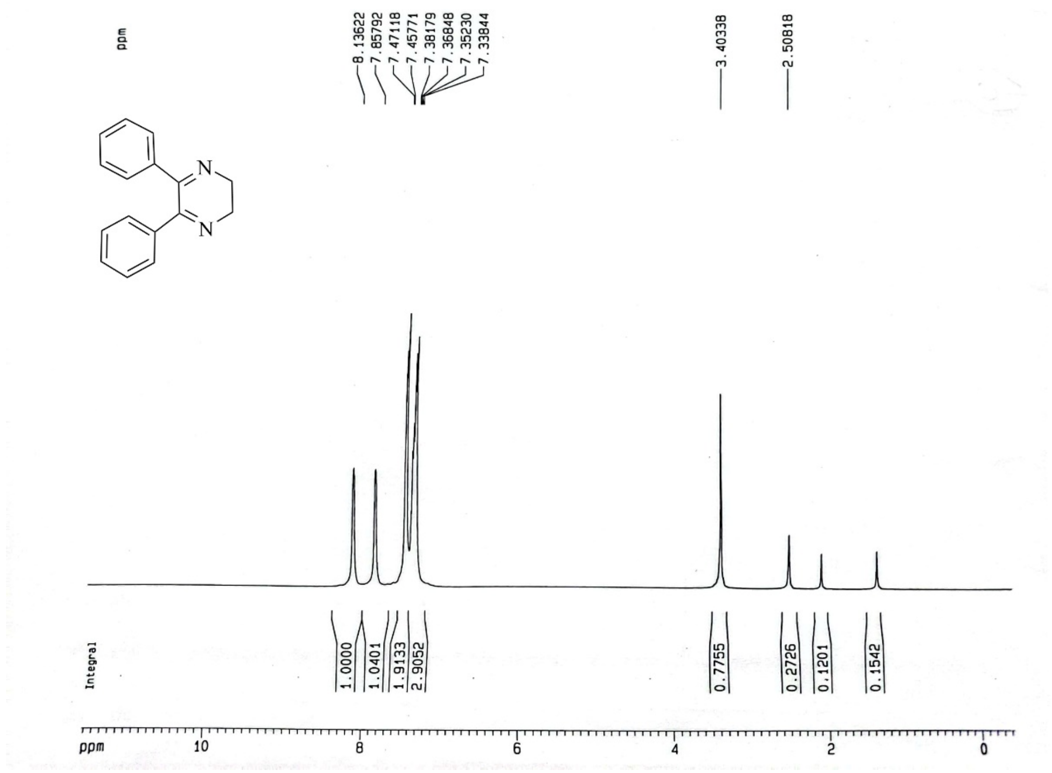

Fig. S15: ^{1}HNMR of 5,6-diphenyl-2,3-dihydropyrazine

5,6-diphenyl-2,3-dihydropyrazine :

^{1}HNMR (500 MHz, DMSO-d6): 3.83 (s, 3H), 5.83 (s, 1H), 6.65 (s, 1H), 6.66-7.95 (m, 8H), 8.43 (s, 1H),); ^{13}C NMR (125 MHz, DMSO-d6): 54.03, 66.86, 115.43, 115.50, 118.56, 126.30, 128.10, 129.81, 130.77, 147.49, 147.91, 163.13, 165.31.

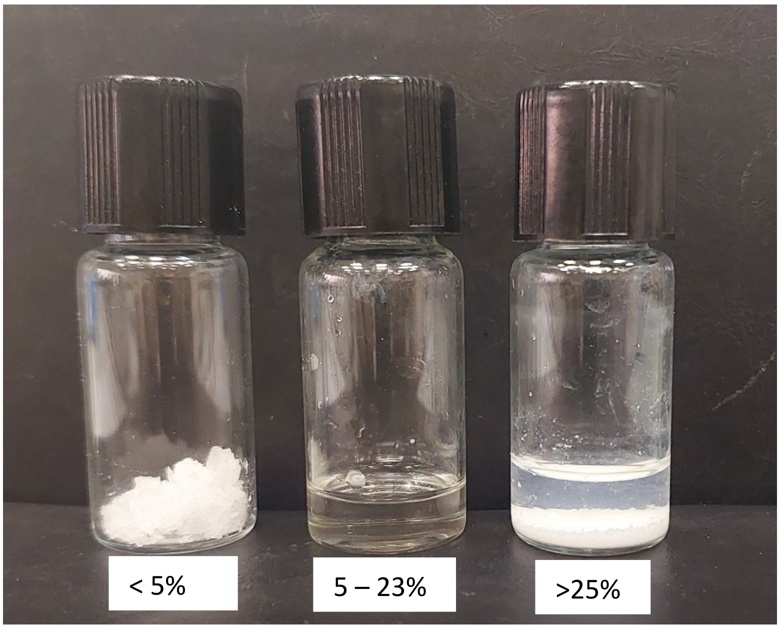

Fig. S16: Water content of DES

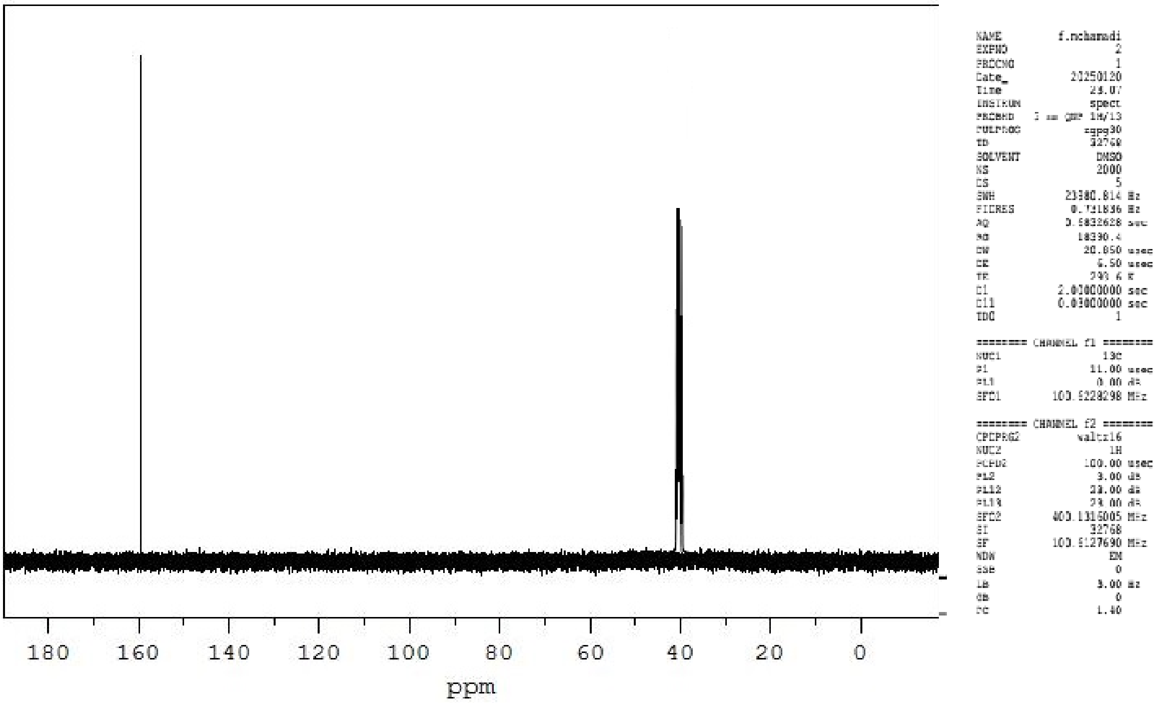

Fig.S17. ^{13}C NMR of urea
